# *O*-Glycoproteomic analysis of engineered heavily glycosylated fusion proteins using nanoHILIC-MS

**DOI:** 10.1007/s00216-022-04318-7

**Published:** 2022-09-22

**Authors:** Gustavo J. Cavallero, Yan Wang, Charles Nwosu, Sheng Gu, Muthuraman Meiyappan, Joseph Zaia

**Affiliations:** 1 Department of Biochemistry, Center for Biomedical Mass Spectrometry, Boston University School of Medicine, Boston, MA 02118 USA; 2 Analytical Development, Pharmaceutical Sciences, Takeda Development Center Americas, Inc., Lexington, MA 02421 USA

**Keywords:** HILIC, Proteoglycans, Chondroitin sulfate, Fusion proteins

## Abstract

Recombinant protein engineering design affects therapeutic properties including protein efficacy, safety, and immunogenicity. Importantly, glycosylation modulates glycoprotein therapeutic pharmacokinetics, pharmacodynamics, and effector functions. Furthermore, the development of fusion proteins requires in-depth characterization of the protein integrity and its glycosylation to evaluate their critical quality attributes. Fc-fusion proteins can be modified by complex glycosylation on the active peptide, the fragment crystallizable (Fc) domain, and the linker peptides. Moreover, the type of glycosylation and the glycan distribution at a given glycosite depend on the host cell line and the expression system conditions that significantly impact safety and efficacy. Because of the inherent heterogeneity of glycosylation, it is necessary to assign glycan structural detail for glycoprotein quality control. Using conventional reversed-phase LC–MS methods, the different glycoforms at a given glycosite elute over a narrow retention time window, and glycopeptide ionization is suppressed by co-eluting non-modified peptides. To overcome this drawback, we used nanoHILIC-MS to characterize the complex glycosylation of UTI-Fc, a fusion protein that greatly increases the half-life of ulinastatin. By this methodology, we identified and characterized ulinastatin glycopeptides at the Fc domain and linker peptide. The results described herein demonstrate the advantages of nanoHILIC-MS to elucidate glycan features on glycotherapeutics that fail to be detected using traditional reversed-phase glycoproteomics.

**Supplementary Information:**

The online version contains supplementary material available at 10.1007/s00216-022-04318-7.

## Introduction

Therapeutic proteins are a primary focus in many pharmaceutical portfolios on account of their efficient treatment of a wide spectrum of diseases including cancers and immune disorders [1]. Fusion proteins have been engineered to optimize the pharmacokinetic and pharmacodynamic properties of the active protein and to enhance Fc domain effector functions or increase the serum half-life [2, 3].

Glycotherapeutics include monoclonal antibodies and recombinant fusion proteins [4]. The latter have received much attention since the finding that fusing biologically active proteins with the fragment of human-derived Ig (including IgG, IgA, IgD, IgM, and IgE) can significantly increase its half-life relative to the original active protein [5]. Fusion proteins are composed of two or more functional domains connected by a linker peptide. The linker peptide maintains cooperative inter-domain interactions that preserve biological activity [6].

Glycosylation strongly influences immunogenicity, half-life, and clinical efficacy of therapeutic proteins [7, 8]. Heterogeneity in glycosylation occurring at glycosites in the active protein, the protein linker, and the immunoglobulin Fc domain has functional and pathological implications [9]. Therefore, precise quantification of glycosylation of a fusion protein is critical for product solubility, stability, pharmacokinetics, pharmacodynamics, bioactivity, immunogenicity, and toxicity [10, 11]. It has been reported that Fc-fusion proteins are modified with *O*-glycans; therefore, a detailed *O*-glycosylation analysis of fusion proteins is required to assure the quality attributes of biotherapeutics [12–15].

The urinary trypsin inhibitor (UTI), known as ulinastatin, is a small acidic chondroitin sulfate proteoglycan that is used to treat acute pancreatitis and has been reported at an increased urinary concentration in many pathological states [16]. In the bloodstream, ulinastatin has a crucial role in anti‐inflammatory responses and is responsible for inhibiting the activities of serine protease enzymes [17]. To increase ulinastatin half-life in serum, bioengineers developed the fusion protein UTI-Fc using the protein linker GGGGS to connect it to the Fc chain. Despite that ulinastatin glycopeptides have been characterized using reversed-phase liquid chromatography–mass spectrometry (LC–MS) [18], there are no previous publications that quantify the distribution of glycoforms and the glycosylation patterns in the fusion protein construct UTI-Fc.

While the use of reversed-phase LC–MS peptide mapping methods is widely applied for characterization analysis of trypsin-digested proteins, the performance of the technique is attenuated during glycopeptide analysis. This is because ionization efficiencies of glycosylated peptides are suppressed by co-eluting unmodified peptides. Moreover, since reversed-phase separations are based on hydrophobic interactions, the glycopeptide glycans shift the retention time relative to the unmodified peptide to a relatively small degree, resulting in the elution of glycopeptides with a common peptide sequence to a narrow retention time window [19]. HILIC, by contrast, exhibits much greater separation of glycopeptides based on interactions of the glycan moieties with the polar stationary phase [20].

In this study, we identified and quantified the relative glycan distribution on UTI-Fc using nanoHILIC-MS for comprehensive mapping *O*-glycopeptides in UTI-Fc fusion protein. We quantified *O*-glycosylated peptides in the active protein domain and describe in detail for the first-time unexpected *O*-glycosylation at the linker and Fc region in UTI-Fc. These results highlight the improved performance of nanoHILIC-MS for the characterization of biotherapeutic protein glycosylation in comparison to RP nanoLC-MS methods.

## Material and methods

### Fusion protein expression

The bioengineered UTI-Fc protein was provided by Takeda biosciences. The fusion protein consisted of a homodimer of two UTI chains, each linked to a human IgG1 Fc chain by a short linker peptide GGGGS.

### Chondroitin sulfate digestion

UTI-Fc samples were buffered and exchanged into 50 mM ammonium bicarbonate, pH 8, using 10 kDa Microcon™ MWCO filters (MilliporeSigma), centrifuged at 8000 g, 4 °C for 3 × 40 min. The samples were digested using 20 mU of chondroitinase ABC (MilliporeSigma) in 25 mM Tris HCl pH 7.5, and 25 mM ammonium acetate at 37 °C overnight. UTI-Fc was recovered subsequently by 3 × 200 μL H_2_O washes through 10 kDa Microcon™ MWCO filters.

### Tryptic glycopeptide mapping

For protein digestion, 10 µg of UTI-Fc was reduced with 10 mM DTT (Sigma) for 30 min at 55 °C. Samples were then alkylated using 50 mM iodoacetamide (ThermoFisher Scientific) for 30 min at room temperature in a dark room. After alkylation, additional DTT was added to quench the IAA. The sample was buffered using 50 mM ammonium bicarbonate and sequencing-grade trypsin (Promega) was added at an enzyme/substrate weight ratio of 1:50 and incubated at 37 °C overnight. Digested samples were desalted using Pierce C18 Spin Columns (Thermo Fischer Scientific). Finally, samples were enriched in glycopeptides using HILIC SPE columns (Hilicon).

### NanoLC analysis

HILIC and RP-LC–MS analyses were performed using a nanoAcquity UPLC system (Waters Corporation) coupled to a Q-Exactive HF mass spectrometer (Thermo Fischer Scientific). The glycopeptide separation was achieved using either a reversed-phase (RP) column (BEH C18, 150 µm × 100 mm Waters Corporation) or a HILIC mode (BEH Amide, 300 µm × 100 mm Waters Corporation). The mobile phase for RP separations consisted of solvent A, 2% ACN with 0.1% FA, and solvent B, 99% ACN with 0.1% FA. RP elution condition consisted of a gradient of 100–60% A in 45 min. The mobile phase for HILIC separations consisted of solvent A, 2% ACN with 0.1% TFA, and solvent B, 99% ACN with 0.1% TFA. HILIC elution conditions consisted of a gradient from 20–75% A in 60 min. The flow rate was set to 0.5 μL/min for RP mode and 0.8 μL/min for HILIC mode. In both, 2–5 μg of the sample was injected and the column temperature was maintained at 40 °C. Two replicates were run in each condition.

### Mass spectrometry analysis

All acquisitions were performed on a Q-Exactive HF mass spectrometer (Thermo Fisher Scientific). Data-dependent acquisition was used in both RP and HILIC analysis in the positive ionization mode for the top 20 most abundant precursor ions. The MS^1^ method settings were as follows: Orbitrap resolution 60,000; mass range 350–2000 m*/z*; automatic gain control 1 × 10^6^; maximum injection time 100 ms. For MS^2^ analysis: Orbitrap resolution 15,000; mass range 200–2000 m*/z*; automatic gain control 1 × 10^5^; maximum injection time 100 ms; normalized collision energy (NCE) 32; dynamic exclusion time of 12 s; isolation window 2.0 m*/z*. Profile data were recorded for both MS1 and MS2 scans.

### Data analysis

Glycopeptide analysis was performed using GlycReSoft 0.4.7 [21]. Glycan search space is included in Supplementary information. Prior to automatic identifications, raw files were converted to mzML format using MSConvert [22] (ProteoWizard version 3.0.11252) with no additional filters. Carbamidomethylation on cysteine residues was set as fixed modification and oxidation on methionine was specified as variable modification. The precursor ion (MS1) mass error tolerance was set to 10 ppm and fragment ion (MS/MS) error tolerance to 20 ppm. We required a minimum of two unique peptides for protein identification. Default parameters were used for setting the threshold score for accepting individual spectra. Glycan structures are represented according to Symbol Nomenclature for Glycans (SNFG) [23] and linkage analysis was not determined, consistent with MIRAGE guidelines [24]. Glycopeptide identifications and scoring function using GlycReSoft are described elsewhere [25]. Briefly, for glycopeptide identification, a scoring function was developed to range between 0 and positive infinity, being based on − 10 * log10(*p* value) of the matched peak intensity against all peaks +  − 10 * log10(*p* value) from a binomial test of the number of matched fragments given the number of theoretical fragments and mass accuracy constraints, weighted by the glycopeptide sequence coverage. This score is further augmented by a small bias towards higher mass accuracy following a Gaussian distribution, and bias towards glycan compositions which contain signature ions present in the matched scan. The glycopeptide false discovery rate is estimated using the target-decoy strategy, and for each glycopeptide, the reported *q*-value is the lowest FDR at which a glycopeptide is accepted. For quantitation analysis, total signal intensity is used. The total signal is an aggregated value from the XICs corresponding to each glycopeptide composition across all charge states and adducts. The total signal is averaged between duplicates for each identified glycopeptide. Standard deviations are also obtained for the set of replicates. The current version of GlycReSoft will not include automatic searches of different types of glycosylation occurring on the same peptide sequence at the same time. This design choice during software development was made because mixing different types of glycosylation (GAG-linker, N-glycans, and O-glycans) leads to unmanageable combinatorial expansion in the glycan search space. As an example, mixing N-glycosylation and O-glycosylation into the same search space as each N-glycopeptide has to have an S or a T by definition, so they would have been combined with O-glycans as well and the combinatorial space turns unmanageable. Multi-glycosylated peptides were found by manually exploring the spectra.

Data accession ID: PXD033645.

## Results and discussion

### HILIC-MS analysis of O-glycosylation at the UTI fragment of UTI-Fc

**Fig. 1:**
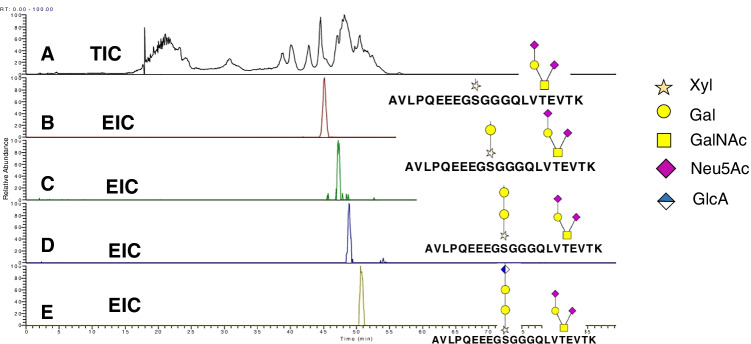
Analysis of UTI-Fc using HILIC nanoLC-MS. Total ion chromatogram (**A**) and extracted ion chromatograms corresponding to the multiple *O*-glycosylated peptide ^1^AVLPQEEEGSGGGQLVTEVTK^21^ indicating the elongation of the CS chain from a single Xyl unit (B), the addition of the first Gal unit (**C**), a second Gal unit (**D**), and the addition of GlcA to complete the CS linker (**E**). Glycan structures are represented according to Symbol Nomenclature for Glycans (SNFG) [23]

To determine *O*-glycosylation sites of the active protein, samples were first digested with chondroitinase ABC to depolymerize the chondroitin sulfate (CS) chains. After depolymerization of the CS chains, samples were trypsin-digested followed by HILIC enrichment of glycopeptides and analyzed using RP and HILIC chromatographic modes, respectively. The nanoHILIC-MS approach allowed us to assign multiple *O*-glycosites on a peptide modified with chondroitin sulfate chains and core 1 *O*-glycans simultaneously with high confidence due to retention time correlation. Figure 1 and Table S1 indicate the glycosylation detected on the CS site at Ser-10 and the presence of mono- and disialylated core 1 *O*-linked glycans on Thr-17. In addition to the expected CS linker saccharide glycopeptides [26], we detected truncated linker saccharides, suggesting that CS chains biosynthesis was incomplete. Remarkably, as shown in Fig. 1, nanoHILIC provided a high confidence assignment of a doubly glycosylated *O*-glycopeptide due to the retention time shifts corresponding to the different glycan structures attached to the modified peptide. As an example, Figure S3 shows the annotated MS/MS spectrum spectra of the peptide ^1^AVLPQEEEGSGGGQLVTEVTK^21^ at the UTI region modified by a truncated chondroitin sulfate chain (xyl-hex_2_) and a core 1 di-sialyalated mucin-type O-glycan (Sial_2_-Hex-HexNac).

**Fig. 2:**
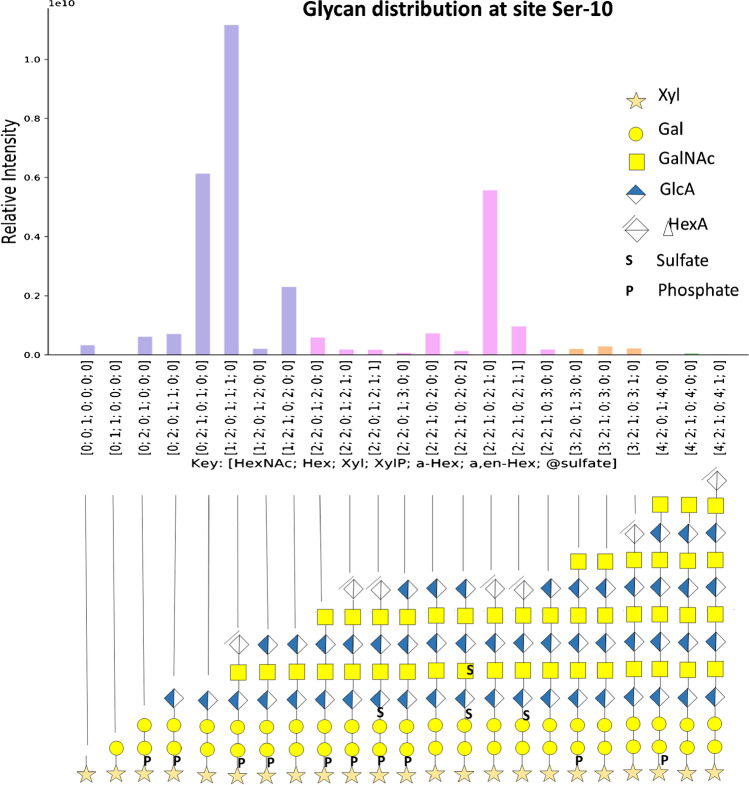
Analysis of UTI-Fc using HILIC nanoLC-MS. CS linker saccharide distribution at glycosite Ser-10. Glycan structures are represented according to Symbol Nomenclature for Glycans (SNFG) [23]

We identified *O*-glycosylation occurring at peptide ^1^AVLPQEEEGSGGGQLVTEVTK^21^ including (i) single site occupancy at S-10, (ii) single site occupancy at T-17, and (iii) simultaneous site occupancy at S-10 and T-17 (Table S1). As the workflow followed in this study involved glycopeptide enrichment, unmodified peptides were not detected and, thus, site occupancy was not addressed. We demonstrated that the CS linker saccharide of the Fab domain in UTI-Fc is highly heterogeneous with respect to glycosylation. We detected Xyl phosphorylation and Gal sulfation in the CS core (Fig. 2). Similar glycoforms for ulinastatin glycosylation have been previously reported from RP-LC–MS analysis [18, 27]. The results show a series of CS linker glycopeptides including those that were cleaved by the chondroitinase enzyme that terminate with a Δ^4,5^-unsaturated uronic acid residue and those that were not cleaved that terminate with a saturated monosaccharide. The latter correspond to biosynthetic CS chain variants for the UTI-Fc.

We compared the performance of nanoHILIC-MS analysis with nanoRP-LC–MS analysis of UTI-Fc. Table S1 summarizes the glycopeptide analysis results using RP and HILIC, and Figure S1 compares the glycopeptide identifications for the different chromatographic modes of analysis.

**Fig. 3:**
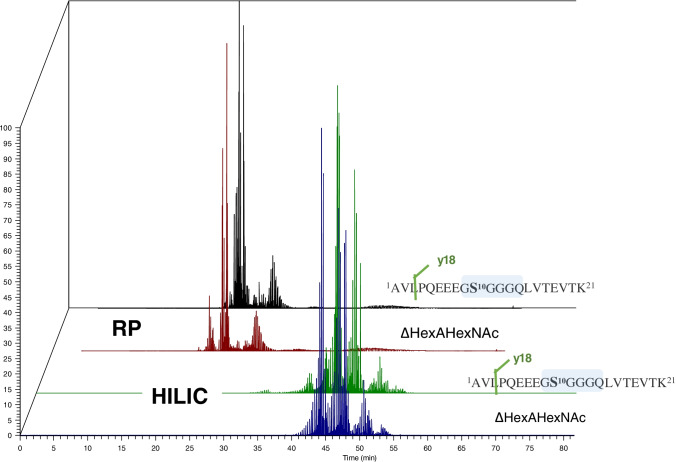
Analysis of UTI-Fc using RP nanoLC-MS and nanoHILIC-MS. Traces: black (y18 most abundant peptide fragmentation in RP analysis), green (y18 most abundant peptide fragmentation in HILIC analysis), red (chondroitin sulfate oxonium reporter ion in RP analysis), blue (chondroitin sulfate oxonium reporter ion in HILIC analysis)

While the most abundant glycoforms at Ser-10 were identified by both LC configurations, RP-LC failed to detect low abundance glycosylation sites on UTI-Fc (Figures S1 and S4 and Tables S1 and S4. This is likely due to signal suppression of co-eluting non-glycopeptides with glycosylated peptides observed during the RP gradient, and to the lower chromatographic resolution of the glycan distribution at a given glycosite [28, 29]. Figure 3 shows the EIC at the MS2 level for the most abundant peptide backbone fragmentation and the chondroitin sulfate oxonium reporter ion, for both reversed-phase and HILIC analysis. In summary, our nanoHILIC approach allowed the assignment of a greater range of glycopeptide glycoforms due to the ability of HILIC to separate the different glycoforms in time, preventing co-elution of non-modified peptides along with glycosylated peptides. Aditionally, we have conducted the analysis of *N*-glycopeptides, showing increased glycoforms identifications using nanoHILIC-MS (Table S4, Figures S9–10).

### Fc O-glycosylation from human IgG–fusion glycoproteins

**Fig. 4:**
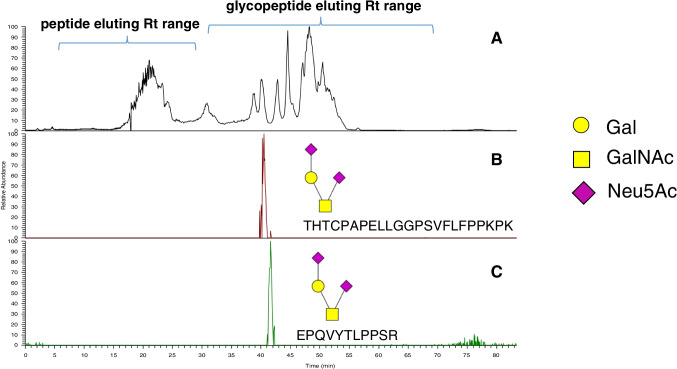
NanoHILIC-MS total ion chromatogram (**A**) and extracted ion chromatograms corresponding to the O-glycopeptides (**B**) ^152^THTCPPCPAPELLGGPSVFLFPPKPK^177^and (**C**) ^274^EPQVYTLPPSR.^284^ modifying the Fc region of UTI-Fc fusion glycoprotein. Glycan structures are represented according to Symbol Nomenclature for Glycans (SNFG)

The IgG Fc is a homodimer connected by an inter-chain disulfide-bond (CH2 domain) and non-covalently paired region (CH3 domains) [30]. It is well-established that the CH2 domains are glycosylated at Asn297 with complex biantennary *N*-glycans [7]. However, *O*-glycoproteomic analyses describing Fc domain *O*-glycosylation and their biological effects in fusion glycoproteins from CHO cell culture are underexplored. In this context, to detect the occurrence of Fc *O*-glycosylation in therapeutic glycoprotein UTI-Fc, we used the nanoHILIC-MS approach to afford in-depth assignment and relative quantification of low abundance glycopeptides. We identified *O*-glycosylation sites in the Fc domain of UTI-Fc glycoprotein on peptides ^274^EPQVYTLPPSR^284^ and ^152^THTCPPCPAPELLGGPSVFLFPPKPK^177^, each substituted with sialylated mucin-type core 1 *O*-glycans (Fig. 4). Figure S5 shows the comparative results using both LC configurations. Notably, these *O*-glycopeptides were identified with high confidence because HILIC resolves glycopeptides based on glycan structures. We observed no co-elution of glycosylated peptides with their non-glycosylated forms, thus, minimizing the occurrence of false positives. We found the relative retention time (*R*_t_) alignment of different glycosylated peptide backbones highly correlated without substantial variation due to the peptide backbone size (less than 1 min *R*_t_ variation) as shown in Fig. 4. The retention time shift is explained due to the higher hydrophobicity of peptide 152–177 bearing the *O*-glycan Hex1-HexNAc1-Neu5Ac2 compared with less hydrophobic peptide 274–284 modified with the same glycan composition. Annotated MS2 spectra of identified glycopeptides are provided in Supplementary Figures S6–S7 and Table S2–S3. By comparison, only Fc domain *O*-glycopeptide 152–177 was assigned using RP nanoLC-MS (Table S1).

### HILIC-MS analysis of O-glycosylation at the linker peptide of UTI-Fc

**Fig. 5:**
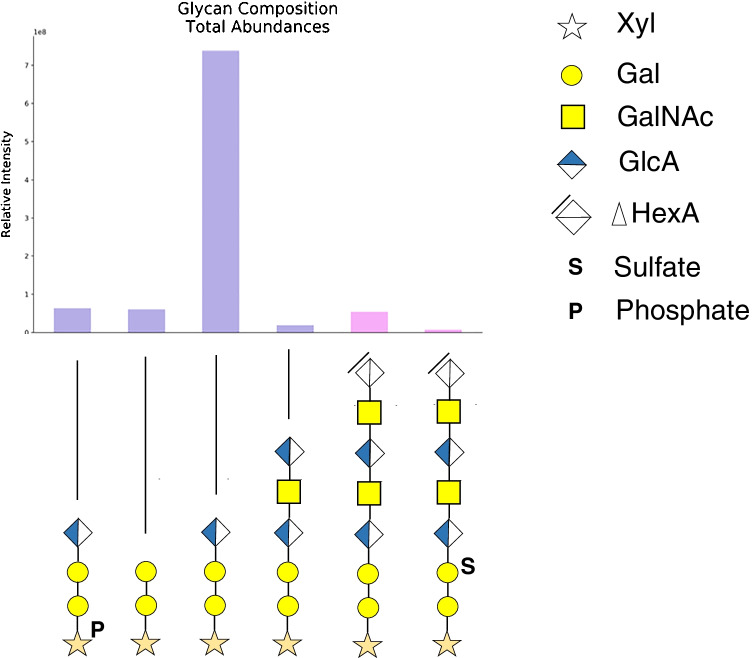
*O*-Glycan distribution modifying the linker GS4 of UTI-Fc. Glycan structures are represented according to Symbol Nomenclature for Glycans (SNFG)

The engineering design of fusion proteins entails connecting active proteins to the Fc moiety (usually IgG1 Fc) using a peptide linker. The most frequently used peptide linkers consist of repeating units of Gly-Gly-Gly-Gly-Ser, (G4S)n. These linkers offer structural flexibility and resist the actions of in vivo proteases [31]. However, it has been reported previously that *O*-xylosylation can occur to Ser residues in G4S linkers [12, 32–34]. In this context, we used nanoHILIC-MS to reveal possible glycosylation at the linker of UTI-Fc. Our HILIC approach allowed us to confidently assign a set of linker saccharide variants at Ser-145 consistent with the incomplete biosynthetic extension of the chondroitin sulfate chains. Figure 5 shows the most abundant *O*-linked glycan distribution modifying the linker peptide GS4. We detected extended CS chains cleaved by chondroitinase digestion to produce the CS core tetrasaccharide plus a ΔHexA-GalNAc unit [18, 26]. The fact that we detected the unsaturated hexasaccharide glycopeptides from the HILIC-MS analysis is consistent with the presence of extended CS chains at this position. It has been reported that the xylosylation level in fusion protein linkers depends not only on the number of G4S repeating unit motifs in the linker saccharide, but also on the 3-dimensional protein structure necessary to expose (G4S)n motifs to the xylosyltransferase enzyme that initiates glycosaminoglycan chain biosynthesis [34, 35]. As shown in Figure S4 and Table S1, by comparison, the analysis of the same sample using RP nanoLC-MS resulted in fewer confident glycoform assignments at CS glycosite Ser-145.

## Conclusions

In this study, we found that a nanoHILIC-MS approach to characterize the complex glycosylation of the fusion protein UTI-FC resolves simultaneously singly and multiply *O*-glycosylated peptides. We showed the influence of glycan structure on the chromatographic retention behavior and that the peptide size only slightly affected the elution profile. Moreover, nanoHILIC produces consistent retention times for shared glycan structures on different peptides, prevents the co-elution of non-glycosylated peptides from glycosylated peptides, and affords higher assignment confidence compared to RP nanoLC-MS *O*-glycoproteomics. In this context, we confidently assigned low abundance glycopeptides on the fusion protein UTI-Fc that escaped assignment using RP nanoLC-MS. Since glycans modify the active protein, the IgG1 Fc, and the linker used for the protein construct, we demonstrate that nanoHILIC provides more comprehensive profiling that may potentiate the ability to determine lot-to-lot variability in protein glycosylation that is important for protein stability, bioactivity, and immunogenicity during the first stages of protein engineering design for therapeutic glycoproteins.

## Supplementary Information

Below is the link to the electronic supplementary material.

Supplementary file1 (DOCX 547 kb)
