# Supplementary material for: O-Glycoproteomic analysis of engineered heavily glycosylated fusion proteins using nanoHILIC-MS
Source: Anal Bioanal Chem. 2022 Sep 22;414(27):7855–63. doi: 10.1007/s00216-022-04318-7 (PMC9568489; doi:10.1007/s00216-022-04318-7)
Supplement: Supplementary file 1 — Supplementary file1 (DOCX 547 kb) [file 216_2022_4318_MOESM1_ESM.docx]

Supplementary Information

*O*-Glycoproteomic Analysis of Engineered Heavily-Glycosylated Fusion Proteins using NanoHILIC-MS

Gustavo J. Cavallero^1^, Yan Wang^2^, Charles Nwosu^2^, Sheng Gu^2^, Muthu Meiyappan^2^, and Joseph Zaia^1*^

^1^ Department of Biochemistry, Center for Biomedical Mass Spectrometry, Boston University School of Medicine, Boston, Massachusetts 02118, United States

^2^ Analytical Development, Pharmaceutical Sciences, Takeda Pharmaceutical Company, Lexington, MA 02421, United States

* Corresponding Author

ORCID

Joseph Zaia: 0000-0001-9497-8701

Tel.: 617-358-2429. E-mail: [jzaia@bu.edu](mailto:jzaia@bu.edu)

Index:

p.4

Figure S1: Glycan distribution at Ser-10 results using nanoHILIC-MS vs reversed-phase nanoLC-MS

p.5

Figure S2: Reverse phase nano-LC-MS analysis of UTI-Fc. A) TIC. Retention time alignment of non-modified peptide B) ^130^EYCGVPGDGDEELLGSGGGGDK^151^, C) ^1^AVLPQEEEGSGGGQLVTEVTK^21^, and D) chondroitin sulfate reporter ion showing the glycan distribution associated to the glycoforms modifying peptide 1-21 and 130-151.

p.6

Figure S3: Annotated spectra corresponding to di-glycosylated peptide ^1^AVLPQEEEGSGGGQLVTEVTK^21^ at the UTI region modified by a truncated chondroitin sulfate chain and a Core 1 di-sialyalated mucin type *O*-glycan.

Figure S4: Glycan distribution at Ser-145 results using nanoHILIC-MS and reversed-phase nanoLC-MS

p.7

Figure S5: Glycan distribution on peptide ^152^TH**T**CPPCPAPELLGGPSVFLFPPKPK^177^.

Figure S6: Annotated spectrum for glycopeptide ^152^THTCPPCPAPELLGGPSVFLFPPKPK^177^ substituted with core 1 *O*-linked glycan structure (Hex1-HexNAc1-Neu5Ac2)

p.8

Figure S7: Annotated spectrum for glycopeptide ^274^ EEQYNSTYR^284^ substituted with core 1 *O*-linked glycan structure (Hex1-HexNAc1-Neu5Ac2).

Figure S8: Glycan distribution at Ser-145 results using nanoHILIC-MS and revered-phase nanoLC-MS

p.9

Table S1: Summary of glycopeptide identified in UTI-Fc using nanoHILIC-MS and reversed-phase LC-MS

p.10:

Table S2: Annotated spectrum for glycopeptide ^152^TH**T**CPPCPAPELLGGPSVFLFPPKPK^177^ substituted with core 1 *O*-linked glycan structure (Hex1-HexNAc1-Neu5Ac2)

p.11

Table S3: Annotated spectrum for glycopeptide ^274^EPQVYTLPPSR^284^ substituted with core 1 *O*-linked glycan structure (Hex1-HexNAc1-Neu5Ac2)

p. 12

Figure S9: Glycan distribution at Asn-226 results using HILIC-MS and RP-MS

p.13

Figure S10: Glycan distribution at Asn-45 results using HILIC-MS and RP-MS

Table S4: Summary of *N*-glycopeptides glycopeptide identified in UTI-Fc using HILIC-MS and RP-MS

p.15

Table S5. List of glycans included as a search space for automatic glycopeptide identification

Figure S1: Glycan distribution at Ser-10 results using A) nanoHILIC-MS B) reversed phase nanoLC-MS

Figure S2: Reverse phase nano-LC-MS analysis of UTI-Fc. A) TIC. Retention time alignment of non-modified peptide B) ^130^EYCGVPGDGDEELLGSGGGGDK^151^, C) ^1^AVLPQEEEGSGGGQLVTEVTK^21^, and D) chondroitin sulfate reporter ion showing the glycan distribution associated to the glycoforms modifying peptide 1-21 and 130-151.

**
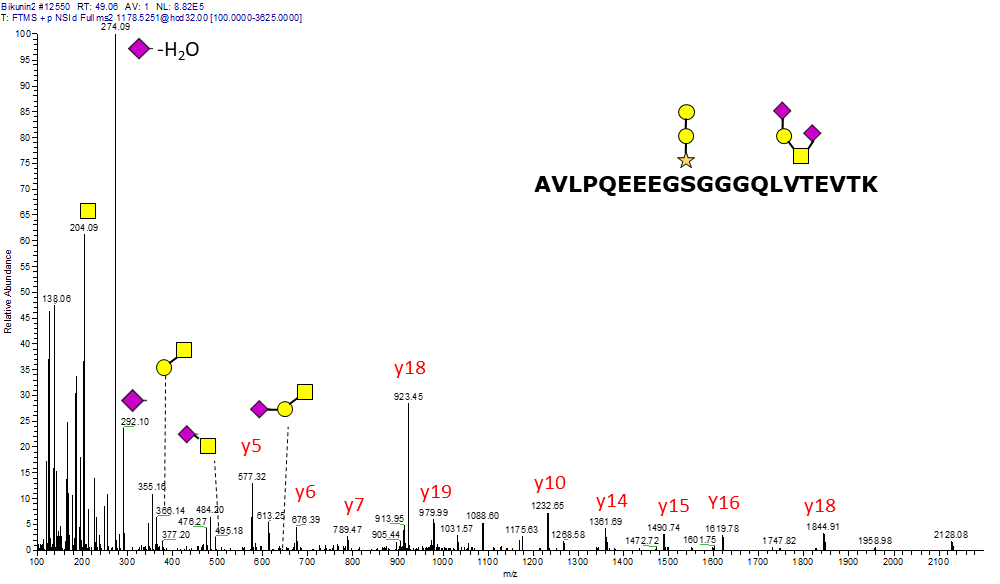
**

Figure S3: Annotated spectra corresponding to di-glycosylated peptide ^1^AVLPQEEEGSGGGQLVTEVTK^21^ at the UTI region modified by a truncated chondroitin sulfate chain and a Core 1 di-sialyalated mucin type *O*-glycan.

Figure S4: Glycan distribution at Ser-145 results using A) nanoHILIC-MS B) reversed phase nanoLC-MS

Figure S5: Glycan distribution on peptide ^152^TH**T**CPPCPAPELLGGPSVFLFPPKPK^177^. Thr-154 is marked as a potential glycosylation site.

Figure S6: Annotated spectrum for glycopeptide ^152^THTCPPCPAPELLGGPSVFLFPPKPK^177^ substituted with core 1 *O*-linked glycan structure (Hex1-HexNAc1-Neu5Ac2)

Figure S7: Annotated spectrum for glycopeptide ^274^ EPQVYTLPPSR^284^ substituted with core 1 *O*-linked glycan structure (Hex1-HexNAc1-Neu5Ac2)

Figure S8: Glycan distribution at Ser-145 results using nanoHILIC-MS and revered-phase nanoLC-MS

Table S1: Summary of glycopeptide identified in UTI-Fc using nanoHILIC-MS and reversed-phase LC-MS


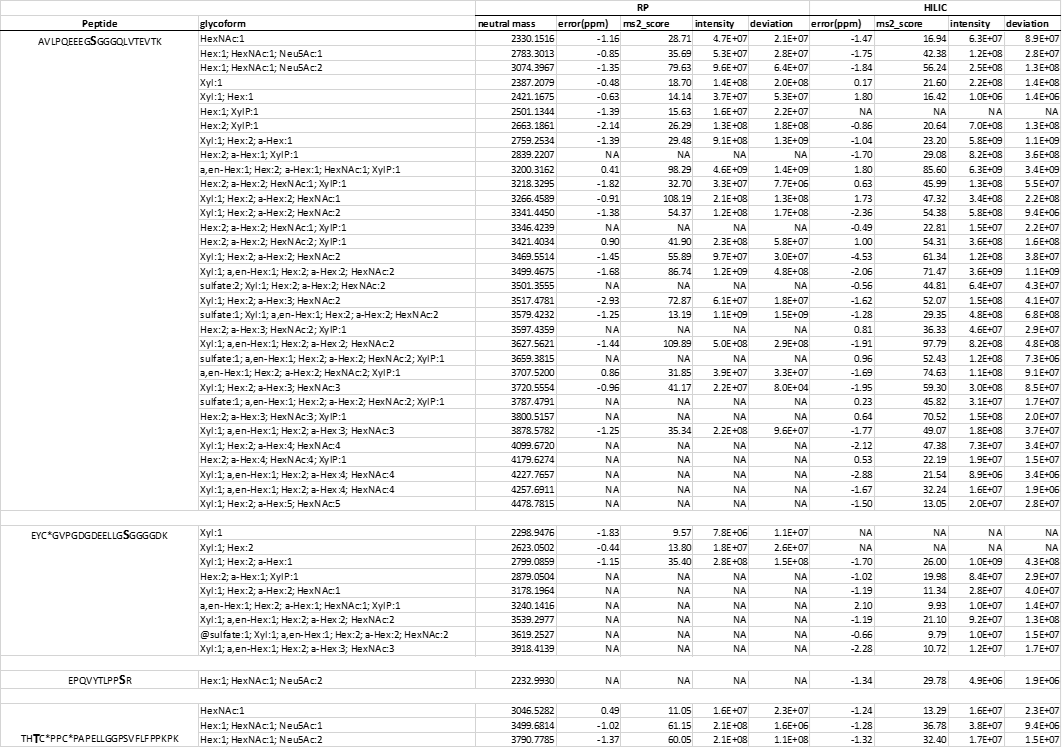


Table S2: Annotated spectrum for glycopeptide ^152^TH**T**CPPCPAPELLGGPSVFLFPPKPK^177^ substituted with core 1 *O*-linked glycan structure (Hex1-HexNAc1-Neu5Ac2)


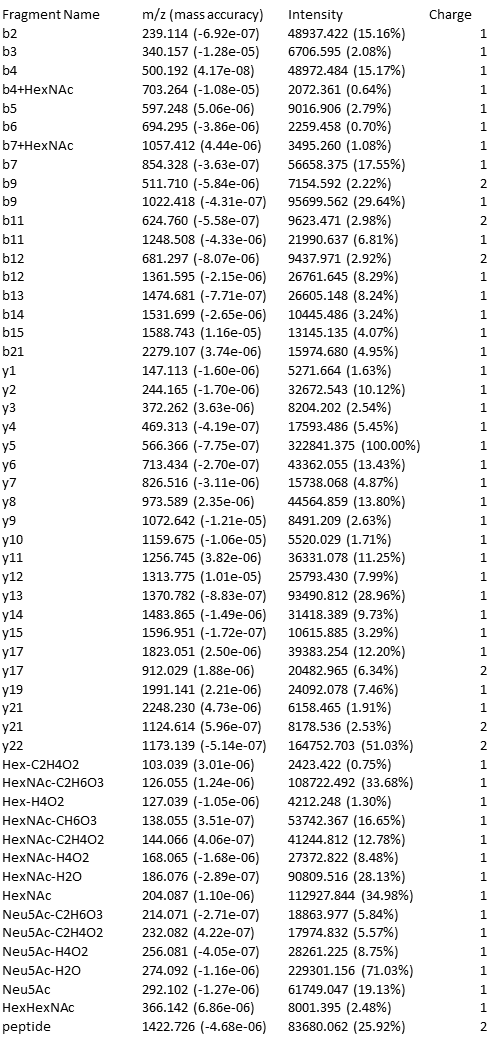


Table S3: Annotated spectrum for glycopeptide ^274^EPQVYTLPPSR^284^ substituted with core 1 O-linked glycan structure (Hex1-HexNAc1-Neu5Ac2)


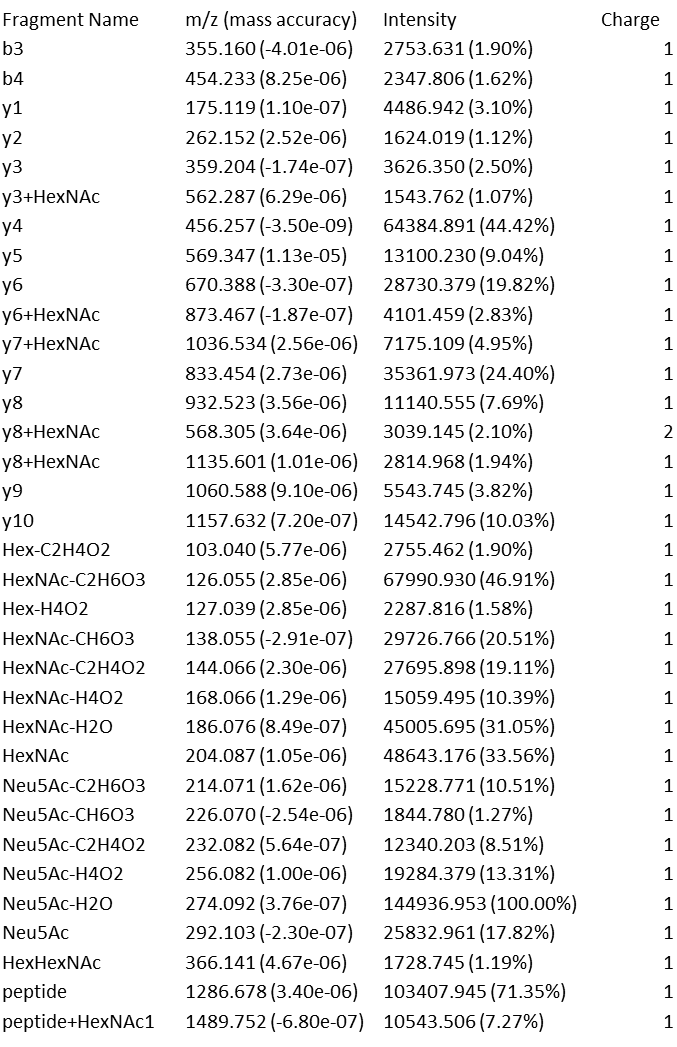


Figure S9: Glycan distribution at Asn-226 results using nanoHILIC-MS vs reversed-phase nanoLC-MS

Figure S10: Glycan distribution at Asn-45 results using HILIC-MS and RP-MS

Table S4: Summary of *N*-glycopeptides glycopeptide identified in UTI-Fc using HILIC-MS and RP-MS

Table S5. List of glycans included as a search space for automatic glycopeptide identification

{HexNAc:1} O-Glycan

{HexNAc:1; Neu5Ac:1} O-Glycan

{HexNAc:1; Neu5Gc:1} O-Glycan

{Hex:1; HexNAc:1} O-Glycan

{Hex:1; HexNAc:1; Neu5Ac:1} O-Glycan

{Hex:1; HexNAc:1; Neu5Ac:2} O-Glycan

{Hex:1; HexNAc:1; Neu5Gc:1} O-Glycan

{Hex:1; HexNAc:1; Neu5Gc:2} O-Glycan

{Xyl:1} GAG-Linker

{Xyl:1; Hex:1} GAG-Linker

{Xyl:1; Hex:2} GAG-Linker

{Xyl:1; Hex:2; a-Hex:1} GAG-Linker

{Xyl:1; Hex:2; a-Hex:2; HexNAc:1} GAG-Linker

{Xyl:1; Hex:2; a-Hex:2; HexNAc:2} GAG-Linker

{Xyl:1; Hex:2; a-Hex:3; HexNAc:2} GAG-Linker

{Xyl:1; Hex:2; a-Hex:3; HexNAc:3} GAG-Linker

{Xyl:1; Hex:2; a-Hex:4; HexNAc:3} GAG-Linker

{Xyl:1; Hex:2; a-Hex:4; HexNAc:4} GAG-Linker

{Xyl:1; Hex:2; a-Hex:5; HexNAc:4} GAG-Linker

{Xyl:1; Hex:2; a-Hex:5; HexNAc:5} GAG-Linker

{Xyl:1; Hex:2; a-Hex:6; HexNAc:5} GAG-Linker

{Xyl:1; Hex:2; a-Hex:6; HexNAc:6} GAG-Linker

{Xyl:1; Hex:2; a-Hex:7; HexNAc:6} GAG-Linker

{Xyl:1; Hex:2; a-Hex:7; HexNAc:7} GAG-Linker

{Xyl:1; Hex:2; a-Hex:8; HexNAc:7} GAG-Linker

{Xyl:1; Hex:2; a-Hex:8; HexNAc:8} GAG-Linker

{XylP:1} GAG-Linker

{XylP:1; Hex:1} GAG-Linker

{XylP:1; Hex:2} GAG-Linker

{XylP:1; Hex:2; a-Hex:1} GAG-Linker

{XylP:1; Hex:2; a-Hex:2; HexNAc:1} GAG-Linker

{XylP:1; Hex:2; a-Hex:2; HexNAc:2} GAG-Linker

{XylP:1; Hex:2; a-Hex:3; HexNAc:2} GAG-Linker

{XylP:1; Hex:2; a-Hex:3; HexNAc:3} GAG-Linker

{XylP:1; Hex:2; a-Hex:4; HexNAc:3} GAG-Linker

{XylP:1; Hex:2; a-Hex:4; HexNAc:4} GAG-Linker

{XylP:1; Hex:2; a-Hex:5; HexNAc:4} GAG-Linker

{XylP:1; Hex:2; a-Hex:5; HexNAc:5} GAG-Linker

{XylP:1; Hex:2; a-Hex:6; HexNAc:5} GAG-Linker

{XylP:1; Hex:2; a-Hex:6; HexNAc:6} GAG-Linker

{XylP:1; Hex:2; a-Hex:7; HexNAc:6} GAG-Linker

{XylP:1; Hex:2; a-Hex:7; HexNAc:7} GAG-Linker

{XylP:1; Hex:2; a-Hex:8; HexNAc:7} GAG-Linker

{XylP:1; Hex:2; a-Hex:8; HexNAc:8} GAG-Linker

{Xyl:1; Hex:2; a-Hex:2; HexNAc:2; Neu5Ac:1} GAG-Linker

{Xyl:1; Hex:2; a-Hex:3; HexNAc:2; Neu5Ac:1} GAG-Linker

{Xyl:1; Hex:2; a-Hex:3; HexNAc:3; Neu5Ac:1} GAG-Linker

{Xyl:1; Hex:2; a-Hex:4; HexNAc:3; Neu5Ac:1} GAG-Linker

{Xyl:1; Hex:2; a-Hex:4; HexNAc:4; Neu5Ac:1} GAG-Linker

{Xyl:1; Hex:2; a-Hex:5; HexNAc:4; Neu5Ac:1} GAG-Linker

{Xyl:1; Hex:2; a-Hex:5; HexNAc:5; Neu5Ac:1} GAG-Linker

{Xyl:1; Hex:2; a-Hex:6; HexNAc:5; Neu5Ac:1} GAG-Linker

{Xyl:1; Hex:2; a-Hex:6; HexNAc:6; Neu5Ac:1} GAG-Linker

{Xyl:1; Hex:2; a-Hex:7; HexNAc:6; Neu5Ac:1} GAG-Linker

{Xyl:1; Hex:2; a-Hex:7; HexNAc:7; Neu5Ac:1} GAG-Linker

{Xyl:1; Hex:2; a-Hex:8; HexNAc:7; Neu5Ac:1} GAG-Linker

{Xyl:1; Hex:2; a-Hex:8; HexNAc:8; Neu5Ac:1} GAG-Linker

{Xyl:1; Hex:2; a-Hex:2; HexNAc:2; Neu5Gc:1} GAG-Linker

{Xyl:1; Hex:2; a-Hex:3; HexNAc:2; Neu5Gc:1} GAG-Linker

{Xyl:1; Hex:2; a-Hex:3; HexNAc:3; Neu5Gc:1} GAG-Linker

{Xyl:1; Hex:2; a-Hex:4; HexNAc:3; Neu5Gc:1} GAG-Linker

{Xyl:1; Hex:2; a-Hex:4; HexNAc:4; Neu5Gc:1} GAG-Linker

{Xyl:1; Hex:2; a-Hex:5; HexNAc:4; Neu5Gc:1} GAG-Linker

{Xyl:1; Hex:2; a-Hex:5; HexNAc:5; Neu5Gc:1} GAG-Linker

{Xyl:1; Hex:2; a-Hex:6; HexNAc:5; Neu5Gc:1} GAG-Linker

{Xyl:1; Hex:2; a-Hex:6; HexNAc:6; Neu5Gc:1} GAG-Linker

{Xyl:1; Hex:2; a-Hex:7; HexNAc:6; Neu5Gc:1} GAG-Linker

{Xyl:1; Hex:2; a-Hex:7; HexNAc:7; Neu5Gc:1} GAG-Linker

{Xyl:1; Hex:2; a-Hex:8; HexNAc:7; Neu5Gc:1} GAG-Linker

{Xyl:1; Hex:2; a-Hex:8; HexNAc:8; Neu5Gc:1} GAG-Linker

{XylP:1; Hex:2; a-Hex:2; HexNAc:2; Neu5Ac:1} GAG-Linker

{XylP:1; Hex:2; a-Hex:3; HexNAc:2; Neu5Ac:1} GAG-Linker

{XylP:1; Hex:2; a-Hex:3; HexNAc:3; Neu5Ac:1} GAG-Linker

{XylP:1; Hex:2; a-Hex:4; HexNAc:3; Neu5Ac:1} GAG-Linker

{XylP:1; Hex:2; a-Hex:4; HexNAc:4; Neu5Ac:1} GAG-Linker

{XylP:1; Hex:2; a-Hex:5; HexNAc:4; Neu5Ac:1} GAG-Linker

{XylP:1; Hex:2; a-Hex:5; HexNAc:5; Neu5Ac:1} GAG-Linker

{XylP:1; Hex:2; a-Hex:6; HexNAc:5; Neu5Ac:1} GAG-Linker

{XylP:1; Hex:2; a-Hex:6; HexNAc:6; Neu5Ac:1} GAG-Linker

{XylP:1; Hex:2; a-Hex:7; HexNAc:6; Neu5Ac:1} GAG-Linker

{XylP:1; Hex:2; a-Hex:7; HexNAc:7; Neu5Ac:1} GAG-Linker

{XylP:1; Hex:2; a-Hex:8; HexNAc:7; Neu5Ac:1} GAG-Linker

{XylP:1; Hex:2; a-Hex:8; HexNAc:8; Neu5Ac:1} GAG-Linker

{XylP:1; Hex:2; a-Hex:2; HexNAc:2; Neu5Gc:1} GAG-Linker

{XylP:1; Hex:2; a-Hex:3; HexNAc:2; Neu5Gc:1} GAG-Linker

{XylP:1; Hex:2; a-Hex:3; HexNAc:3; Neu5Gc:1} GAG-Linker

{XylP:1; Hex:2; a-Hex:4; HexNAc:3; Neu5Gc:1} GAG-Linker

{XylP:1; Hex:2; a-Hex:4; HexNAc:4; Neu5Gc:1} GAG-Linker

{XylP:1; Hex:2; a-Hex:5; HexNAc:4; Neu5Gc:1} GAG-Linker

{XylP:1; Hex:2; a-Hex:5; HexNAc:5; Neu5Gc:1} GAG-Linker

{XylP:1; Hex:2; a-Hex:6; HexNAc:5; Neu5Gc:1} GAG-Linker

{XylP:1; Hex:2; a-Hex:6; HexNAc:6; Neu5Gc:1} GAG-Linker

{XylP:1; Hex:2; a-Hex:7; HexNAc:6; Neu5Gc:1} GAG-Linker

{XylP:1; Hex:2; a-Hex:7; HexNAc:7; Neu5Gc:1} GAG-Linker

{XylP:1; Hex:2; a-Hex:8; HexNAc:7; Neu5Gc:1} GAG-Linker

{XylP:1; Hex:2; a-Hex:8; HexNAc:8; Neu5Gc:1} GAG-Linker

{Xyl:1; @sulfate:1} GAG-Linker

{Xyl:1; Hex:1; @sulfate:1} GAG-Linker

{Xyl:1; Hex:2; @sulfate:1} GAG-Linker

{Xyl:1; Hex:2; a-Hex:1; @sulfate:1} GAG-Linker

{Xyl:1; Hex:2; a-Hex:2; HexNAc:1; @sulfate:1} GAG-Linker

{Xyl:1; Hex:2; a-Hex:2; HexNAc:2; @sulfate:1} GAG-Linker

{Xyl:1; Hex:2; a-Hex:3; HexNAc:2; @sulfate:1} GAG-Linker

{Xyl:1; Hex:2; a-Hex:3; HexNAc:3; @sulfate:1} GAG-Linker

{Xyl:1; Hex:2; a-Hex:4; HexNAc:3; @sulfate:1} GAG-Linker

{Xyl:1; Hex:2; a-Hex:4; HexNAc:4; @sulfate:1} GAG-Linker

{Xyl:1; Hex:2; a-Hex:5; HexNAc:4; @sulfate:1} GAG-Linker

{Xyl:1; Hex:2; a-Hex:5; HexNAc:5; @sulfate:1} GAG-Linker

{Xyl:1; Hex:2; a-Hex:6; HexNAc:5; @sulfate:1} GAG-Linker

{Xyl:1; Hex:2; a-Hex:6; HexNAc:6; @sulfate:1} GAG-Linker

{Xyl:1; Hex:2; a-Hex:7; HexNAc:6; @sulfate:1} GAG-Linker

{Xyl:1; Hex:2; a-Hex:7; HexNAc:7; @sulfate:1} GAG-Linker

{Xyl:1; Hex:2; a-Hex:8; HexNAc:7; @sulfate:1} GAG-Linker

{Xyl:1; Hex:2; a-Hex:8; HexNAc:8; @sulfate:1} GAG-Linker

{XylP:1; @sulfate:1} GAG-Linker

{XylP:1; Hex:1; @sulfate:1} GAG-Linker

{XylP:1; Hex:2; @sulfate:1} GAG-Linker

{XylP:1; Hex:2; a-Hex:1; @sulfate:1} GAG-Linker

{XylP:1; Hex:2; a-Hex:2; HexNAc:1; @sulfate:1} GAG-Linker

{XylP:1; Hex:2; a-Hex:2; HexNAc:2; @sulfate:1} GAG-Linker

{XylP:1; Hex:2; a-Hex:3; HexNAc:2; @sulfate:1} GAG-Linker

{XylP:1; Hex:2; a-Hex:3; HexNAc:3; @sulfate:1} GAG-Linker

{XylP:1; Hex:2; a-Hex:4; HexNAc:3; @sulfate:1} GAG-Linker

{XylP:1; Hex:2; a-Hex:4; HexNAc:4; @sulfate:1} GAG-Linker

{XylP:1; Hex:2; a-Hex:5; HexNAc:4; @sulfate:1} GAG-Linker

{XylP:1; Hex:2; a-Hex:5; HexNAc:5; @sulfate:1} GAG-Linker

{XylP:1; Hex:2; a-Hex:6; HexNAc:5; @sulfate:1} GAG-Linker

{XylP:1; Hex:2; a-Hex:6; HexNAc:6; @sulfate:1} GAG-Linker

{XylP:1; Hex:2; a-Hex:7; HexNAc:6; @sulfate:1} GAG-Linker

{XylP:1; Hex:2; a-Hex:7; HexNAc:7; @sulfate:1} GAG-Linker

{XylP:1; Hex:2; a-Hex:8; HexNAc:7; @sulfate:1} GAG-Linker

{XylP:1; Hex:2; a-Hex:8; HexNAc:8; @sulfate:1} GAG-Linker

{Xyl:1; Hex:2; a-Hex:2; HexNAc:2; Neu5Ac:1; @sulfate:1} GAG-Linker

{Xyl:1; Hex:2; a-Hex:3; HexNAc:2; Neu5Ac:1; @sulfate:1} GAG-Linker

{Xyl:1; Hex:2; a-Hex:3; HexNAc:3; Neu5Ac:1; @sulfate:1} GAG-Linker

{Xyl:1; Hex:2; a-Hex:4; HexNAc:3; Neu5Ac:1; @sulfate:1} GAG-Linker

{Xyl:1; Hex:2; a-Hex:4; HexNAc:4; Neu5Ac:1; @sulfate:1} GAG-Linker

{Xyl:1; Hex:2; a-Hex:5; HexNAc:4; Neu5Ac:1; @sulfate:1} GAG-Linker

{Xyl:1; Hex:2; a-Hex:5; HexNAc:5; Neu5Ac:1; @sulfate:1} GAG-Linker

{Xyl:1; Hex:2; a-Hex:6; HexNAc:5; Neu5Ac:1; @sulfate:1} GAG-Linker

{Xyl:1; Hex:2; a-Hex:6; HexNAc:6; Neu5Ac:1; @sulfate:1} GAG-Linker

{Xyl:1; Hex:2; a-Hex:7; HexNAc:6; Neu5Ac:1; @sulfate:1} GAG-Linker

{Xyl:1; Hex:2; a-Hex:7; HexNAc:7; Neu5Ac:1; @sulfate:1} GAG-Linker

{Xyl:1; Hex:2; a-Hex:8; HexNAc:7; Neu5Ac:1; @sulfate:1} GAG-Linker

{Xyl:1; Hex:2; a-Hex:8; HexNAc:8; Neu5Ac:1; @sulfate:1} GAG-Linker

{Xyl:1; Hex:2; a-Hex:2; HexNAc:2; Neu5Gc:1; @sulfate:1} GAG-Linker

{Xyl:1; Hex:2; a-Hex:3; HexNAc:2; Neu5Gc:1; @sulfate:1} GAG-Linker

{Xyl:1; Hex:2; a-Hex:3; HexNAc:3; Neu5Gc:1; @sulfate:1} GAG-Linker

{Xyl:1; Hex:2; a-Hex:4; HexNAc:3; Neu5Gc:1; @sulfate:1} GAG-Linker

{Xyl:1; Hex:2; a-Hex:4; HexNAc:4; Neu5Gc:1; @sulfate:1} GAG-Linker

{Xyl:1; Hex:2; a-Hex:5; HexNAc:4; Neu5Gc:1; @sulfate:1} GAG-Linker

{Xyl:1; Hex:2; a-Hex:5; HexNAc:5; Neu5Gc:1; @sulfate:1} GAG-Linker

{Xyl:1; Hex:2; a-Hex:6; HexNAc:5; Neu5Gc:1; @sulfate:1} GAG-Linker

{Xyl:1; Hex:2; a-Hex:6; HexNAc:6; Neu5Gc:1; @sulfate:1} GAG-Linker

{Xyl:1; Hex:2; a-Hex:7; HexNAc:6; Neu5Gc:1; @sulfate:1} GAG-Linker

{Xyl:1; Hex:2; a-Hex:7; HexNAc:7; Neu5Gc:1; @sulfate:1} GAG-Linker

{Xyl:1; Hex:2; a-Hex:8; HexNAc:7; Neu5Gc:1; @sulfate:1} GAG-Linker

{Xyl:1; Hex:2; a-Hex:8; HexNAc:8; Neu5Gc:1; @sulfate:1} GAG-Linker

{XylP:1; Hex:2; a-Hex:2; HexNAc:2; Neu5Ac:1; @sulfate:1} GAG-Linker

{XylP:1; Hex:2; a-Hex:3; HexNAc:2; Neu5Ac:1; @sulfate:1} GAG-Linker

{XylP:1; Hex:2; a-Hex:3; HexNAc:3; Neu5Ac:1; @sulfate:1} GAG-Linker

{XylP:1; Hex:2; a-Hex:4; HexNAc:3; Neu5Ac:1; @sulfate:1} GAG-Linker

{XylP:1; Hex:2; a-Hex:4; HexNAc:4; Neu5Ac:1; @sulfate:1} GAG-Linker

{XylP:1; Hex:2; a-Hex:5; HexNAc:4; Neu5Ac:1; @sulfate:1} GAG-Linker

{XylP:1; Hex:2; a-Hex:5; HexNAc:5; Neu5Ac:1; @sulfate:1} GAG-Linker

{XylP:1; Hex:2; a-Hex:6; HexNAc:5; Neu5Ac:1; @sulfate:1} GAG-Linker

{XylP:1; Hex:2; a-Hex:6; HexNAc:6; Neu5Ac:1; @sulfate:1} GAG-Linker

{XylP:1; Hex:2; a-Hex:7; HexNAc:6; Neu5Ac:1; @sulfate:1} GAG-Linker

{XylP:1; Hex:2; a-Hex:7; HexNAc:7; Neu5Ac:1; @sulfate:1} GAG-Linker

{XylP:1; Hex:2; a-Hex:8; HexNAc:7; Neu5Ac:1; @sulfate:1} GAG-Linker

{XylP:1; Hex:2; a-Hex:8; HexNAc:8; Neu5Ac:1; @sulfate:1} GAG-Linker

{XylP:1; Hex:2; a-Hex:2; HexNAc:2; Neu5Gc:1; @sulfate:1} GAG-Linker

{XylP:1; Hex:2; a-Hex:3; HexNAc:2; Neu5Gc:1; @sulfate:1} GAG-Linker

{XylP:1; Hex:2; a-Hex:3; HexNAc:3; Neu5Gc:1; @sulfate:1} GAG-Linker

{XylP:1; Hex:2; a-Hex:4; HexNAc:3; Neu5Gc:1; @sulfate:1} GAG-Linker

{XylP:1; Hex:2; a-Hex:4; HexNAc:4; Neu5Gc:1; @sulfate:1} GAG-Linker

{XylP:1; Hex:2; a-Hex:5; HexNAc:4; Neu5Gc:1; @sulfate:1} GAG-Linker

{XylP:1; Hex:2; a-Hex:5; HexNAc:5; Neu5Gc:1; @sulfate:1} GAG-Linker

{XylP:1; Hex:2; a-Hex:6; HexNAc:5; Neu5Gc:1; @sulfate:1} GAG-Linker

{XylP:1; Hex:2; a-Hex:6; HexNAc:6; Neu5Gc:1; @sulfate:1} GAG-Linker

{XylP:1; Hex:2; a-Hex:7; HexNAc:6; Neu5Gc:1; @sulfate:1} GAG-Linker

{XylP:1; Hex:2; a-Hex:7; HexNAc:7; Neu5Gc:1; @sulfate:1} GAG-Linker

{XylP:1; Hex:2; a-Hex:8; HexNAc:7; Neu5Gc:1; @sulfate:1} GAG-Linker

{XylP:1; Hex:2; a-Hex:8; HexNAc:8; Neu5Gc:1; @sulfate:1} GAG-Linker

{Xyl:1; @sulfate:2} GAG-Linker

{Xyl:1; Hex:1; @sulfate:2} GAG-Linker

{Xyl:1; Hex:2; @sulfate:2} GAG-Linker

{Xyl:1; Hex:2; a-Hex:1; @sulfate:2} GAG-Linker

{Xyl:1; Hex:2; a-Hex:2; HexNAc:1; @sulfate:2} GAG-Linker

{Xyl:1; Hex:2; a-Hex:2; HexNAc:2; @sulfate:2} GAG-Linker

{Xyl:1; Hex:2; a-Hex:3; HexNAc:2; @sulfate:2} GAG-Linker

{Xyl:1; Hex:2; a-Hex:3; HexNAc:3; @sulfate:2} GAG-Linker

{Xyl:1; Hex:2; a-Hex:4; HexNAc:3; @sulfate:2} GAG-Linker

{Xyl:1; Hex:2; a-Hex:4; HexNAc:4; @sulfate:2} GAG-Linker

{Xyl:1; Hex:2; a-Hex:5; HexNAc:4; @sulfate:2} GAG-Linker

{Xyl:1; Hex:2; a-Hex:5; HexNAc:5; @sulfate:2} GAG-Linker

{Xyl:1; Hex:2; a-Hex:6; HexNAc:5; @sulfate:2} GAG-Linker

{Xyl:1; Hex:2; a-Hex:6; HexNAc:6; @sulfate:2} GAG-Linker

{Xyl:1; Hex:2; a-Hex:7; HexNAc:6; @sulfate:2} GAG-Linker

{Xyl:1; Hex:2; a-Hex:7; HexNAc:7; @sulfate:2} GAG-Linker

{Xyl:1; Hex:2; a-Hex:8; HexNAc:7; @sulfate:2} GAG-Linker

{Xyl:1; Hex:2; a-Hex:8; HexNAc:8; @sulfate:2} GAG-Linker

{XylP:1; @sulfate:2} GAG-Linker

{XylP:1; Hex:1; @sulfate:2} GAG-Linker

{XylP:1; Hex:2; @sulfate:2} GAG-Linker

{XylP:1; Hex:2; a-Hex:1; @sulfate:2} GAG-Linker

{XylP:1; Hex:2; a-Hex:2; HexNAc:1; @sulfate:2} GAG-Linker

{XylP:1; Hex:2; a-Hex:2; HexNAc:2; @sulfate:2} GAG-Linker

{XylP:1; Hex:2; a-Hex:3; HexNAc:2; @sulfate:2} GAG-Linker

{XylP:1; Hex:2; a-Hex:3; HexNAc:3; @sulfate:2} GAG-Linker

{XylP:1; Hex:2; a-Hex:4; HexNAc:3; @sulfate:2} GAG-Linker

{XylP:1; Hex:2; a-Hex:4; HexNAc:4; @sulfate:2} GAG-Linker

{XylP:1; Hex:2; a-Hex:5; HexNAc:4; @sulfate:2} GAG-Linker

{XylP:1; Hex:2; a-Hex:5; HexNAc:5; @sulfate:2} GAG-Linker

{XylP:1; Hex:2; a-Hex:6; HexNAc:5; @sulfate:2} GAG-Linker

{XylP:1; Hex:2; a-Hex:6; HexNAc:6; @sulfate:2} GAG-Linker

{XylP:1; Hex:2; a-Hex:7; HexNAc:6; @sulfate:2} GAG-Linker

{XylP:1; Hex:2; a-Hex:7; HexNAc:7; @sulfate:2} GAG-Linker

{XylP:1; Hex:2; a-Hex:8; HexNAc:7; @sulfate:2} GAG-Linker

{XylP:1; Hex:2; a-Hex:8; HexNAc:8; @sulfate:2} GAG-Linker

{Xyl:1; Hex:2; a-Hex:2; HexNAc:2; Neu5Ac:1; @sulfate:2} GAG-Linker

{Xyl:1; Hex:2; a-Hex:3; HexNAc:2; Neu5Ac:1; @sulfate:2} GAG-Linker

{Xyl:1; Hex:2; a-Hex:3; HexNAc:3; Neu5Ac:1; @sulfate:2} GAG-Linker

{Xyl:1; Hex:2; a-Hex:4; HexNAc:3; Neu5Ac:1; @sulfate:2} GAG-Linker

{Xyl:1; Hex:2; a-Hex:4; HexNAc:4; Neu5Ac:1; @sulfate:2} GAG-Linker

{Xyl:1; Hex:2; a-Hex:5; HexNAc:4; Neu5Ac:1; @sulfate:2} GAG-Linker

{Xyl:1; Hex:2; a-Hex:5; HexNAc:5; Neu5Ac:1; @sulfate:2} GAG-Linker

{Xyl:1; Hex:2; a-Hex:6; HexNAc:5; Neu5Ac:1; @sulfate:2} GAG-Linker

{Xyl:1; Hex:2; a-Hex:6; HexNAc:6; Neu5Ac:1; @sulfate:2} GAG-Linker

{Xyl:1; Hex:2; a-Hex:7; HexNAc:6; Neu5Ac:1; @sulfate:2} GAG-Linker

{Xyl:1; Hex:2; a-Hex:7; HexNAc:7; Neu5Ac:1; @sulfate:2} GAG-Linker

{Xyl:1; Hex:2; a-Hex:8; HexNAc:7; Neu5Ac:1; @sulfate:2} GAG-Linker

{Xyl:1; Hex:2; a-Hex:8; HexNAc:8; Neu5Ac:1; @sulfate:2} GAG-Linker

{Xyl:1; Hex:2; a-Hex:2; HexNAc:2; Neu5Gc:1; @sulfate:2} GAG-Linker

{Xyl:1; Hex:2; a-Hex:3; HexNAc:2; Neu5Gc:1; @sulfate:2} GAG-Linker

{Xyl:1; Hex:2; a-Hex:3; HexNAc:3; Neu5Gc:1; @sulfate:2} GAG-Linker

{Xyl:1; Hex:2; a-Hex:4; HexNAc:3; Neu5Gc:1; @sulfate:2} GAG-Linker

{Xyl:1; Hex:2; a-Hex:4; HexNAc:4; Neu5Gc:1; @sulfate:2} GAG-Linker

{Xyl:1; Hex:2; a-Hex:5; HexNAc:4; Neu5Gc:1; @sulfate:2} GAG-Linker

{Xyl:1; Hex:2; a-Hex:5; HexNAc:5; Neu5Gc:1; @sulfate:2} GAG-Linker

{Xyl:1; Hex:2; a-Hex:6; HexNAc:5; Neu5Gc:1; @sulfate:2} GAG-Linker

{Xyl:1; Hex:2; a-Hex:6; HexNAc:6; Neu5Gc:1; @sulfate:2} GAG-Linker

{Xyl:1; Hex:2; a-Hex:7; HexNAc:6; Neu5Gc:1; @sulfate:2} GAG-Linker

{Xyl:1; Hex:2; a-Hex:7; HexNAc:7; Neu5Gc:1; @sulfate:2} GAG-Linker

{Xyl:1; Hex:2; a-Hex:8; HexNAc:7; Neu5Gc:1; @sulfate:2} GAG-Linker

{Xyl:1; Hex:2; a-Hex:8; HexNAc:8; Neu5Gc:1; @sulfate:2} GAG-Linker

{XylP:1; Hex:2; a-Hex:2; HexNAc:2; Neu5Ac:1; @sulfate:2} GAG-Linker

{XylP:1; Hex:2; a-Hex:3; HexNAc:2; Neu5Ac:1; @sulfate:2} GAG-Linker

{XylP:1; Hex:2; a-Hex:3; HexNAc:3; Neu5Ac:1; @sulfate:2} GAG-Linker

{XylP:1; Hex:2; a-Hex:4; HexNAc:3; Neu5Ac:1; @sulfate:2} GAG-Linker

{XylP:1; Hex:2; a-Hex:4; HexNAc:4; Neu5Ac:1; @sulfate:2} GAG-Linker

{XylP:1; Hex:2; a-Hex:5; HexNAc:4; Neu5Ac:1; @sulfate:2} GAG-Linker

{XylP:1; Hex:2; a-Hex:5; HexNAc:5; Neu5Ac:1; @sulfate:2} GAG-Linker

{XylP:1; Hex:2; a-Hex:6; HexNAc:5; Neu5Ac:1; @sulfate:2} GAG-Linker

{XylP:1; Hex:2; a-Hex:6; HexNAc:6; Neu5Ac:1; @sulfate:2} GAG-Linker

{XylP:1; Hex:2; a-Hex:7; HexNAc:6; Neu5Ac:1; @sulfate:2} GAG-Linker

{XylP:1; Hex:2; a-Hex:7; HexNAc:7; Neu5Ac:1; @sulfate:2} GAG-Linker

{XylP:1; Hex:2; a-Hex:8; HexNAc:7; Neu5Ac:1; @sulfate:2} GAG-Linker

{XylP:1; Hex:2; a-Hex:8; HexNAc:8; Neu5Ac:1; @sulfate:2} GAG-Linker

{XylP:1; Hex:2; a-Hex:2; HexNAc:2; Neu5Gc:1; @sulfate:2} GAG-Linker

{XylP:1; Hex:2; a-Hex:3; HexNAc:2; Neu5Gc:1; @sulfate:2} GAG-Linker

{XylP:1; Hex:2; a-Hex:3; HexNAc:3; Neu5Gc:1; @sulfate:2} GAG-Linker

{XylP:1; Hex:2; a-Hex:4; HexNAc:3; Neu5Gc:1; @sulfate:2} GAG-Linker

{XylP:1; Hex:2; a-Hex:4; HexNAc:4; Neu5Gc:1; @sulfate:2} GAG-Linker

{XylP:1; Hex:2; a-Hex:5; HexNAc:4; Neu5Gc:1; @sulfate:2} GAG-Linker

{XylP:1; Hex:2; a-Hex:5; HexNAc:5; Neu5Gc:1; @sulfate:2} GAG-Linker

{XylP:1; Hex:2; a-Hex:6; HexNAc:5; Neu5Gc:1; @sulfate:2} GAG-Linker

{XylP:1; Hex:2; a-Hex:6; HexNAc:6; Neu5Gc:1; @sulfate:2} GAG-Linker

{XylP:1; Hex:2; a-Hex:7; HexNAc:6; Neu5Gc:1; @sulfate:2} GAG-Linker

{XylP:1; Hex:2; a-Hex:7; HexNAc:7; Neu5Gc:1; @sulfate:2} GAG-Linker

{XylP:1; Hex:2; a-Hex:8; HexNAc:7; Neu5Gc:1; @sulfate:2} GAG-Linker

{XylP:1; Hex:2; a-Hex:8; HexNAc:8; Neu5Gc:1; @sulfate:2} GAG-Linker

{Xyl:1; @sulfate:3} GAG-Linker

{Xyl:1; Hex:1; @sulfate:3} GAG-Linker

{Xyl:1; Hex:2; @sulfate:3} GAG-Linker

{Xyl:1; Hex:2; a-Hex:1; @sulfate:3} GAG-Linker

{Xyl:1; Hex:2; a-Hex:2; HexNAc:1; @sulfate:3} GAG-Linker

{Xyl:1; Hex:2; a-Hex:2; HexNAc:2; @sulfate:3} GAG-Linker

{Xyl:1; Hex:2; a-Hex:3; HexNAc:2; @sulfate:3} GAG-Linker

{Xyl:1; Hex:2; a-Hex:3; HexNAc:3; @sulfate:3} GAG-Linker

{Xyl:1; Hex:2; a-Hex:4; HexNAc:3; @sulfate:3} GAG-Linker

{Xyl:1; Hex:2; a-Hex:4; HexNAc:4; @sulfate:3} GAG-Linker

{Xyl:1; Hex:2; a-Hex:5; HexNAc:4; @sulfate:3} GAG-Linker

{Xyl:1; Hex:2; a-Hex:5; HexNAc:5; @sulfate:3} GAG-Linker

{Xyl:1; Hex:2; a-Hex:6; HexNAc:5; @sulfate:3} GAG-Linker

{Xyl:1; Hex:2; a-Hex:6; HexNAc:6; @sulfate:3} GAG-Linker

{Xyl:1; Hex:2; a-Hex:7; HexNAc:6; @sulfate:3} GAG-Linker

{Xyl:1; Hex:2; a-Hex:7; HexNAc:7; @sulfate:3} GAG-Linker

{Xyl:1; Hex:2; a-Hex:8; HexNAc:7; @sulfate:3} GAG-Linker

{Xyl:1; Hex:2; a-Hex:8; HexNAc:8; @sulfate:3} GAG-Linker

{XylP:1; @sulfate:3} GAG-Linker

{XylP:1; Hex:1; @sulfate:3} GAG-Linker

{XylP:1; Hex:2; @sulfate:3} GAG-Linker

{XylP:1; Hex:2; a-Hex:1; @sulfate:3} GAG-Linker

{XylP:1; Hex:2; a-Hex:2; HexNAc:1; @sulfate:3} GAG-Linker

{XylP:1; Hex:2; a-Hex:2; HexNAc:2; @sulfate:3} GAG-Linker

{XylP:1; Hex:2; a-Hex:3; HexNAc:2; @sulfate:3} GAG-Linker

{XylP:1; Hex:2; a-Hex:3; HexNAc:3; @sulfate:3} GAG-Linker

{XylP:1; Hex:2; a-Hex:4; HexNAc:3; @sulfate:3} GAG-Linker

{XylP:1; Hex:2; a-Hex:4; HexNAc:4; @sulfate:3} GAG-Linker

{XylP:1; Hex:2; a-Hex:5; HexNAc:4; @sulfate:3} GAG-Linker

{XylP:1; Hex:2; a-Hex:5; HexNAc:5; @sulfate:3} GAG-Linker

{XylP:1; Hex:2; a-Hex:6; HexNAc:5; @sulfate:3} GAG-Linker

{XylP:1; Hex:2; a-Hex:6; HexNAc:6; @sulfate:3} GAG-Linker

{XylP:1; Hex:2; a-Hex:7; HexNAc:6; @sulfate:3} GAG-Linker

{XylP:1; Hex:2; a-Hex:7; HexNAc:7; @sulfate:3} GAG-Linker

{XylP:1; Hex:2; a-Hex:8; HexNAc:7; @sulfate:3} GAG-Linker

{XylP:1; Hex:2; a-Hex:8; HexNAc:8; @sulfate:3} GAG-Linker

{Xyl:1; Hex:2; a-Hex:2; HexNAc:2; Neu5Ac:1; @sulfate:3} GAG-Linker

{Xyl:1; Hex:2; a-Hex:3; HexNAc:2; Neu5Ac:1; @sulfate:3} GAG-Linker

{Xyl:1; Hex:2; a-Hex:3; HexNAc:3; Neu5Ac:1; @sulfate:3} GAG-Linker

{Xyl:1; Hex:2; a-Hex:4; HexNAc:3; Neu5Ac:1; @sulfate:3} GAG-Linker

{Xyl:1; Hex:2; a-Hex:4; HexNAc:4; Neu5Ac:1; @sulfate:3} GAG-Linker

{Xyl:1; Hex:2; a-Hex:5; HexNAc:4; Neu5Ac:1; @sulfate:3} GAG-Linker

{Xyl:1; Hex:2; a-Hex:5; HexNAc:5; Neu5Ac:1; @sulfate:3} GAG-Linker

{Xyl:1; Hex:2; a-Hex:6; HexNAc:5; Neu5Ac:1; @sulfate:3} GAG-Linker

{Xyl:1; Hex:2; a-Hex:6; HexNAc:6; Neu5Ac:1; @sulfate:3} GAG-Linker

{Xyl:1; Hex:2; a-Hex:7; HexNAc:6; Neu5Ac:1; @sulfate:3} GAG-Linker

{Xyl:1; Hex:2; a-Hex:7; HexNAc:7; Neu5Ac:1; @sulfate:3} GAG-Linker

{Xyl:1; Hex:2; a-Hex:8; HexNAc:7; Neu5Ac:1; @sulfate:3} GAG-Linker

{Xyl:1; Hex:2; a-Hex:8; HexNAc:8; Neu5Ac:1; @sulfate:3} GAG-Linker

{Xyl:1; Hex:2; a-Hex:2; HexNAc:2; Neu5Gc:1; @sulfate:3} GAG-Linker

{Xyl:1; Hex:2; a-Hex:3; HexNAc:2; Neu5Gc:1; @sulfate:3} GAG-Linker

{Xyl:1; Hex:2; a-Hex:3; HexNAc:3; Neu5Gc:1; @sulfate:3} GAG-Linker

{Xyl:1; Hex:2; a-Hex:4; HexNAc:3; Neu5Gc:1; @sulfate:3} GAG-Linker

{Xyl:1; Hex:2; a-Hex:4; HexNAc:4; Neu5Gc:1; @sulfate:3} GAG-Linker

{Xyl:1; Hex:2; a-Hex:5; HexNAc:4; Neu5Gc:1; @sulfate:3} GAG-Linker

{Xyl:1; Hex:2; a-Hex:5; HexNAc:5; Neu5Gc:1; @sulfate:3} GAG-Linker

{Xyl:1; Hex:2; a-Hex:6; HexNAc:5; Neu5Gc:1; @sulfate:3} GAG-Linker

{Xyl:1; Hex:2; a-Hex:6; HexNAc:6; Neu5Gc:1; @sulfate:3} GAG-Linker

{Xyl:1; Hex:2; a-Hex:7; HexNAc:6; Neu5Gc:1; @sulfate:3} GAG-Linker

{Xyl:1; Hex:2; a-Hex:7; HexNAc:7; Neu5Gc:1; @sulfate:3} GAG-Linker

{Xyl:1; Hex:2; a-Hex:8; HexNAc:7; Neu5Gc:1; @sulfate:3} GAG-Linker

{Xyl:1; Hex:2; a-Hex:8; HexNAc:8; Neu5Gc:1; @sulfate:3} GAG-Linker

{XylP:1; Hex:2; a-Hex:2; HexNAc:2; Neu5Ac:1; @sulfate:3} GAG-Linker

{XylP:1; Hex:2; a-Hex:3; HexNAc:2; Neu5Ac:1; @sulfate:3} GAG-Linker

{XylP:1; Hex:2; a-Hex:3; HexNAc:3; Neu5Ac:1; @sulfate:3} GAG-Linker

{XylP:1; Hex:2; a-Hex:4; HexNAc:3; Neu5Ac:1; @sulfate:3} GAG-Linker

{XylP:1; Hex:2; a-Hex:4; HexNAc:4; Neu5Ac:1; @sulfate:3} GAG-Linker

{XylP:1; Hex:2; a-Hex:5; HexNAc:4; Neu5Ac:1; @sulfate:3} GAG-Linker

{XylP:1; Hex:2; a-Hex:5; HexNAc:5; Neu5Ac:1; @sulfate:3} GAG-Linker

{XylP:1; Hex:2; a-Hex:6; HexNAc:5; Neu5Ac:1; @sulfate:3} GAG-Linker

{XylP:1; Hex:2; a-Hex:6; HexNAc:6; Neu5Ac:1; @sulfate:3} GAG-Linker

{XylP:1; Hex:2; a-Hex:7; HexNAc:6; Neu5Ac:1; @sulfate:3} GAG-Linker

{XylP:1; Hex:2; a-Hex:7; HexNAc:7; Neu5Ac:1; @sulfate:3} GAG-Linker

{XylP:1; Hex:2; a-Hex:8; HexNAc:7; Neu5Ac:1; @sulfate:3} GAG-Linker

{XylP:1; Hex:2; a-Hex:8; HexNAc:8; Neu5Ac:1; @sulfate:3} GAG-Linker

{XylP:1; Hex:2; a-Hex:2; HexNAc:2; Neu5Gc:1; @sulfate:3} GAG-Linker

{XylP:1; Hex:2; a-Hex:3; HexNAc:2; Neu5Gc:1; @sulfate:3} GAG-Linker

{XylP:1; Hex:2; a-Hex:3; HexNAc:3; Neu5Gc:1; @sulfate:3} GAG-Linker

{XylP:1; Hex:2; a-Hex:4; HexNAc:3; Neu5Gc:1; @sulfate:3} GAG-Linker

{XylP:1; Hex:2; a-Hex:4; HexNAc:4; Neu5Gc:1; @sulfate:3} GAG-Linker

{XylP:1; Hex:2; a-Hex:5; HexNAc:4; Neu5Gc:1; @sulfate:3} GAG-Linker

{XylP:1; Hex:2; a-Hex:5; HexNAc:5; Neu5Gc:1; @sulfate:3} GAG-Linker

{XylP:1; Hex:2; a-Hex:6; HexNAc:5; Neu5Gc:1; @sulfate:3} GAG-Linker

{XylP:1; Hex:2; a-Hex:6; HexNAc:6; Neu5Gc:1; @sulfate:3} GAG-Linker

{XylP:1; Hex:2; a-Hex:7; HexNAc:6; Neu5Gc:1; @sulfate:3} GAG-Linker

{XylP:1; Hex:2; a-Hex:7; HexNAc:7; Neu5Gc:1; @sulfate:3} GAG-Linker

{XylP:1; Hex:2; a-Hex:8; HexNAc:7; Neu5Gc:1; @sulfate:3} GAG-Linker

{XylP:1; Hex:2; a-Hex:8; HexNAc:8; Neu5Gc:1; @sulfate:3} GAG-Linker

{Xyl:1; @sulfate:4} GAG-Linker

{Xyl:1; Hex:1; @sulfate:4} GAG-Linker

{Xyl:1; Hex:2; @sulfate:4} GAG-Linker

{Xyl:1; Hex:2; a-Hex:1; @sulfate:4} GAG-Linker

{Xyl:1; Hex:2; a-Hex:2; HexNAc:1; @sulfate:4} GAG-Linker

{Xyl:1; Hex:2; a-Hex:2; HexNAc:2; @sulfate:4} GAG-Linker

{Xyl:1; Hex:2; a-Hex:3; HexNAc:2; @sulfate:4} GAG-Linker

{Xyl:1; Hex:2; a-Hex:3; HexNAc:3; @sulfate:4} GAG-Linker

{Xyl:1; Hex:2; a-Hex:4; HexNAc:3; @sulfate:4} GAG-Linker

{Xyl:1; Hex:2; a-Hex:4; HexNAc:4; @sulfate:4} GAG-Linker

{Xyl:1; Hex:2; a-Hex:5; HexNAc:4; @sulfate:4} GAG-Linker

{Xyl:1; Hex:2; a-Hex:5; HexNAc:5; @sulfate:4} GAG-Linker

{Xyl:1; Hex:2; a-Hex:6; HexNAc:5; @sulfate:4} GAG-Linker

{Xyl:1; Hex:2; a-Hex:6; HexNAc:6; @sulfate:4} GAG-Linker

{Xyl:1; Hex:2; a-Hex:7; HexNAc:6; @sulfate:4} GAG-Linker

{Xyl:1; Hex:2; a-Hex:7; HexNAc:7; @sulfate:4} GAG-Linker

{Xyl:1; Hex:2; a-Hex:8; HexNAc:7; @sulfate:4} GAG-Linker

{Xyl:1; Hex:2; a-Hex:8; HexNAc:8; @sulfate:4} GAG-Linker

{XylP:1; @sulfate:4} GAG-Linker

{XylP:1; Hex:1; @sulfate:4} GAG-Linker

{XylP:1; Hex:2; @sulfate:4} GAG-Linker

{XylP:1; Hex:2; a-Hex:1; @sulfate:4} GAG-Linker

{XylP:1; Hex:2; a-Hex:2; HexNAc:1; @sulfate:4} GAG-Linker

{XylP:1; Hex:2; a-Hex:2; HexNAc:2; @sulfate:4} GAG-Linker

{XylP:1; Hex:2; a-Hex:3; HexNAc:2; @sulfate:4} GAG-Linker

{XylP:1; Hex:2; a-Hex:3; HexNAc:3; @sulfate:4} GAG-Linker

{XylP:1; Hex:2; a-Hex:4; HexNAc:3; @sulfate:4} GAG-Linker

{XylP:1; Hex:2; a-Hex:4; HexNAc:4; @sulfate:4} GAG-Linker

{XylP:1; Hex:2; a-Hex:5; HexNAc:4; @sulfate:4} GAG-Linker

{XylP:1; Hex:2; a-Hex:5; HexNAc:5; @sulfate:4} GAG-Linker

{XylP:1; Hex:2; a-Hex:6; HexNAc:5; @sulfate:4} GAG-Linker

{XylP:1; Hex:2; a-Hex:6; HexNAc:6; @sulfate:4} GAG-Linker

{XylP:1; Hex:2; a-Hex:7; HexNAc:6; @sulfate:4} GAG-Linker

{XylP:1; Hex:2; a-Hex:7; HexNAc:7; @sulfate:4} GAG-Linker

{XylP:1; Hex:2; a-Hex:8; HexNAc:7; @sulfate:4} GAG-Linker

{XylP:1; Hex:2; a-Hex:8; HexNAc:8; @sulfate:4} GAG-Linker

{Xyl:1; Hex:2; a-Hex:2; HexNAc:2; Neu5Ac:1; @sulfate:4} GAG-Linker

{Xyl:1; Hex:2; a-Hex:3; HexNAc:2; Neu5Ac:1; @sulfate:4} GAG-Linker

{Xyl:1; Hex:2; a-Hex:3; HexNAc:3; Neu5Ac:1; @sulfate:4} GAG-Linker

{Xyl:1; Hex:2; a-Hex:4; HexNAc:3; Neu5Ac:1; @sulfate:4} GAG-Linker

{Xyl:1; Hex:2; a-Hex:4; HexNAc:4; Neu5Ac:1; @sulfate:4} GAG-Linker

{Xyl:1; Hex:2; a-Hex:5; HexNAc:4; Neu5Ac:1; @sulfate:4} GAG-Linker

{Xyl:1; Hex:2; a-Hex:5; HexNAc:5; Neu5Ac:1; @sulfate:4} GAG-Linker

{Xyl:1; Hex:2; a-Hex:6; HexNAc:5; Neu5Ac:1; @sulfate:4} GAG-Linker

{Xyl:1; Hex:2; a-Hex:6; HexNAc:6; Neu5Ac:1; @sulfate:4} GAG-Linker

{Xyl:1; Hex:2; a-Hex:7; HexNAc:6; Neu5Ac:1; @sulfate:4} GAG-Linker

{Xyl:1; Hex:2; a-Hex:7; HexNAc:7; Neu5Ac:1; @sulfate:4} GAG-Linker

{Xyl:1; Hex:2; a-Hex:8; HexNAc:7; Neu5Ac:1; @sulfate:4} GAG-Linker

{Xyl:1; Hex:2; a-Hex:8; HexNAc:8; Neu5Ac:1; @sulfate:4} GAG-Linker

{Xyl:1; Hex:2; a-Hex:2; HexNAc:2; Neu5Gc:1; @sulfate:4} GAG-Linker

{Xyl:1; Hex:2; a-Hex:3; HexNAc:2; Neu5Gc:1; @sulfate:4} GAG-Linker

{Xyl:1; Hex:2; a-Hex:3; HexNAc:3; Neu5Gc:1; @sulfate:4} GAG-Linker

{Xyl:1; Hex:2; a-Hex:4; HexNAc:3; Neu5Gc:1; @sulfate:4} GAG-Linker

{Xyl:1; Hex:2; a-Hex:4; HexNAc:4; Neu5Gc:1; @sulfate:4} GAG-Linker

{Xyl:1; Hex:2; a-Hex:5; HexNAc:4; Neu5Gc:1; @sulfate:4} GAG-Linker

{Xyl:1; Hex:2; a-Hex:5; HexNAc:5; Neu5Gc:1; @sulfate:4} GAG-Linker

{Xyl:1; Hex:2; a-Hex:6; HexNAc:5; Neu5Gc:1; @sulfate:4} GAG-Linker

{Xyl:1; Hex:2; a-Hex:6; HexNAc:6; Neu5Gc:1; @sulfate:4} GAG-Linker

{Xyl:1; Hex:2; a-Hex:7; HexNAc:6; Neu5Gc:1; @sulfate:4} GAG-Linker

{Xyl:1; Hex:2; a-Hex:7; HexNAc:7; Neu5Gc:1; @sulfate:4} GAG-Linker

{Xyl:1; Hex:2; a-Hex:8; HexNAc:7; Neu5Gc:1; @sulfate:4} GAG-Linker

{Xyl:1; Hex:2; a-Hex:8; HexNAc:8; Neu5Gc:1; @sulfate:4} GAG-Linker

{XylP:1; Hex:2; a-Hex:2; HexNAc:2; Neu5Ac:1; @sulfate:4} GAG-Linker

{XylP:1; Hex:2; a-Hex:3; HexNAc:2; Neu5Ac:1; @sulfate:4} GAG-Linker

{XylP:1; Hex:2; a-Hex:3; HexNAc:3; Neu5Ac:1; @sulfate:4} GAG-Linker

{XylP:1; Hex:2; a-Hex:4; HexNAc:3; Neu5Ac:1; @sulfate:4} GAG-Linker

{XylP:1; Hex:2; a-Hex:4; HexNAc:4; Neu5Ac:1; @sulfate:4} GAG-Linker

{XylP:1; Hex:2; a-Hex:5; HexNAc:4; Neu5Ac:1; @sulfate:4} GAG-Linker

{XylP:1; Hex:2; a-Hex:5; HexNAc:5; Neu5Ac:1; @sulfate:4} GAG-Linker

{XylP:1; Hex:2; a-Hex:6; HexNAc:5; Neu5Ac:1; @sulfate:4} GAG-Linker

{XylP:1; Hex:2; a-Hex:6; HexNAc:6; Neu5Ac:1; @sulfate:4} GAG-Linker

{XylP:1; Hex:2; a-Hex:7; HexNAc:6; Neu5Ac:1; @sulfate:4} GAG-Linker

{XylP:1; Hex:2; a-Hex:7; HexNAc:7; Neu5Ac:1; @sulfate:4} GAG-Linker

{XylP:1; Hex:2; a-Hex:8; HexNAc:7; Neu5Ac:1; @sulfate:4} GAG-Linker

{XylP:1; Hex:2; a-Hex:8; HexNAc:8; Neu5Ac:1; @sulfate:4} GAG-Linker

{XylP:1; Hex:2; a-Hex:2; HexNAc:2; Neu5Gc:1; @sulfate:4} GAG-Linker

{XylP:1; Hex:2; a-Hex:3; HexNAc:2; Neu5Gc:1; @sulfate:4} GAG-Linker

{XylP:1; Hex:2; a-Hex:3; HexNAc:3; Neu5Gc:1; @sulfate:4} GAG-Linker

{XylP:1; Hex:2; a-Hex:4; HexNAc:3; Neu5Gc:1; @sulfate:4} GAG-Linker

{XylP:1; Hex:2; a-Hex:4; HexNAc:4; Neu5Gc:1; @sulfate:4} GAG-Linker

{XylP:1; Hex:2; a-Hex:5; HexNAc:4; Neu5Gc:1; @sulfate:4} GAG-Linker

{XylP:1; Hex:2; a-Hex:5; HexNAc:5; Neu5Gc:1; @sulfate:4} GAG-Linker

{XylP:1; Hex:2; a-Hex:6; HexNAc:5; Neu5Gc:1; @sulfate:4} GAG-Linker

{XylP:1; Hex:2; a-Hex:6; HexNAc:6; Neu5Gc:1; @sulfate:4} GAG-Linker

{XylP:1; Hex:2; a-Hex:7; HexNAc:6; Neu5Gc:1; @sulfate:4} GAG-Linker

{XylP:1; Hex:2; a-Hex:7; HexNAc:7; Neu5Gc:1; @sulfate:4} GAG-Linker

{XylP:1; Hex:2; a-Hex:8; HexNAc:7; Neu5Gc:1; @sulfate:4} GAG-Linker

{XylP:1; Hex:2; a-Hex:8; HexNAc:8; Neu5Gc:1; @sulfate:4} GAG-Linker

{Xyl:1; Hex:2; a-Hex:2; HexNAc:2; a,en-Hex:1} GAG-Linker

{Xyl:1; Hex:2; a-Hex:3; HexNAc:3; a,en-Hex:1} GAG-Linker

{Xyl:1; Hex:2; a-Hex:4; HexNAc:4; a,en-Hex:1} GAG-Linker

{Xyl:1; Hex:2; a-Hex:5; HexNAc:5; a,en-Hex:1} GAG-Linker

{Xyl:1; Hex:2; a-Hex:6; HexNAc:6; a,en-Hex:1} GAG-Linker

{Xyl:1; Hex:2; a-Hex:7; HexNAc:7; a,en-Hex:1} GAG-Linker

{XylP:1; Hex:2; a-Hex:1; HexNAc:1; a,en-Hex:1} GAG-Linker

{XylP:1; Hex:2; a-Hex:2; HexNAc:2; a,en-Hex:1} GAG-Linker

{XylP:1; Hex:2; a-Hex:3; HexNAc:3; a,en-Hex:1} GAG-Linker

{XylP:1; Hex:2; a-Hex:4; HexNAc:4; a,en-Hex:1} GAG-Linker

{XylP:1; Hex:2; a-Hex:5; HexNAc:5; a,en-Hex:1} GAG-Linker

{XylP:1; Hex:2; a-Hex:6; HexNAc:6; a,en-Hex:1} GAG-Linker

{XylP:1; Hex:2; a-Hex:7; HexNAc:7; a,en-Hex:1} GAG-Linker

{Xyl:1; Hex:2; a-Hex:2; HexNAc:2; Neu5Ac:1; a,en-Hex:1} GAG-Linker

{Xyl:1; Hex:2; a-Hex:3; HexNAc:3; Neu5Ac:1; a,en-Hex:1} GAG-Linker

{Xyl:1; Hex:2; a-Hex:4; HexNAc:4; Neu5Ac:1; a,en-Hex:1} GAG-Linker

{Xyl:1; Hex:2; a-Hex:5; HexNAc:5; Neu5Ac:1; a,en-Hex:1} GAG-Linker

{Xyl:1; Hex:2; a-Hex:6; HexNAc:6; Neu5Ac:1; a,en-Hex:1} GAG-Linker

{Xyl:1; Hex:2; a-Hex:7; HexNAc:7; Neu5Ac:1; a,en-Hex:1} GAG-Linker

{Xyl:1; Hex:2; a-Hex:2; HexNAc:2; Neu5Gc:1; a,en-Hex:1} GAG-Linker

{Xyl:1; Hex:2; a-Hex:3; HexNAc:3; Neu5Gc:1; a,en-Hex:1} GAG-Linker

{Xyl:1; Hex:2; a-Hex:4; HexNAc:4; Neu5Gc:1; a,en-Hex:1} GAG-Linker

{Xyl:1; Hex:2; a-Hex:5; HexNAc:5; Neu5Gc:1; a,en-Hex:1} GAG-Linker

{Xyl:1; Hex:2; a-Hex:6; HexNAc:6; Neu5Gc:1; a,en-Hex:1} GAG-Linker

{Xyl:1; Hex:2; a-Hex:7; HexNAc:7; Neu5Gc:1; a,en-Hex:1} GAG-Linker

{XylP:1; Hex:2; a-Hex:2; HexNAc:2; Neu5Ac:1; a,en-Hex:1} GAG-Linker

{XylP:1; Hex:2; a-Hex:3; HexNAc:3; Neu5Ac:1; a,en-Hex:1} GAG-Linker

{XylP:1; Hex:2; a-Hex:4; HexNAc:4; Neu5Ac:1; a,en-Hex:1} GAG-Linker

{XylP:1; Hex:2; a-Hex:5; HexNAc:5; Neu5Ac:1; a,en-Hex:1} GAG-Linker

{XylP:1; Hex:2; a-Hex:6; HexNAc:6; Neu5Ac:1; a,en-Hex:1} GAG-Linker

{XylP:1; Hex:2; a-Hex:7; HexNAc:7; Neu5Ac:1; a,en-Hex:1} GAG-Linker

{XylP:1; Hex:2; a-Hex:2; HexNAc:2; Neu5Gc:1; a,en-Hex:1} GAG-Linker

{XylP:1; Hex:2; a-Hex:3; HexNAc:3; Neu5Gc:1; a,en-Hex:1} GAG-Linker

{XylP:1; Hex:2; a-Hex:4; HexNAc:4; Neu5Gc:1; a,en-Hex:1} GAG-Linker

{XylP:1; Hex:2; a-Hex:5; HexNAc:5; Neu5Gc:1; a,en-Hex:1} GAG-Linker

{XylP:1; Hex:2; a-Hex:6; HexNAc:6; Neu5Gc:1; a,en-Hex:1} GAG-Linker

{XylP:1; Hex:2; a-Hex:7; HexNAc:7; Neu5Gc:1; a,en-Hex:1} GAG-Linker

{Xyl:1; Hex:2; a-Hex:2; HexNAc:2; @sulfate:1; a,en-Hex:1} GAG-Linker

{Xyl:1; Hex:2; a-Hex:3; HexNAc:3; @sulfate:1; a,en-Hex:1} GAG-Linker

{Xyl:1; Hex:2; a-Hex:4; HexNAc:4; @sulfate:1; a,en-Hex:1} GAG-Linker

{Xyl:1; Hex:2; a-Hex:5; HexNAc:5; @sulfate:1; a,en-Hex:1} GAG-Linker

{Xyl:1; Hex:2; a-Hex:6; HexNAc:6; @sulfate:1; a,en-Hex:1} GAG-Linker

{Xyl:1; Hex:2; a-Hex:7; HexNAc:7; @sulfate:1; a,en-Hex:1} GAG-Linker

{Xyl:1; Hex:2; a-Hex:8; HexNAc:8; @sulfate:1; a,en-Hex:1} GAG-Linker

{XylP:1; Hex:2; a-Hex:2; HexNAc:2; @sulfate:1; a,en-Hex:1} GAG-Linker

{XylP:1; Hex:2; a-Hex:3; HexNAc:3; @sulfate:1; a,en-Hex:1} GAG-Linker

{XylP:1; Hex:2; a-Hex:4; HexNAc:4; @sulfate:1; a,en-Hex:1} GAG-Linker

{XylP:1; Hex:2; a-Hex:5; HexNAc:5; @sulfate:1; a,en-Hex:1} GAG-Linker

{XylP:1; Hex:2; a-Hex:6; HexNAc:6; @sulfate:1; a,en-Hex:1} GAG-Linker

{XylP:1; Hex:2; a-Hex:7; HexNAc:7; @sulfate:1; a,en-Hex:1} GAG-Linker

{XylP:1; Hex:2; a-Hex:8; HexNAc:8; @sulfate:1; a,en-Hex:1} GAG-Linker

{Xyl:1; Hex:2; a-Hex:2; HexNAc:2; Neu5Ac:1; @sulfate:1; a,en-Hex:1} GAG-Linker

{Xyl:1; Hex:2; a-Hex:3; HexNAc:3; Neu5Ac:1; @sulfate:1; a,en-Hex:1} GAG-Linker

{Xyl:1; Hex:2; a-Hex:4; HexNAc:4; Neu5Ac:1; @sulfate:1; a,en-Hex:1} GAG-Linker

{Xyl:1; Hex:2; a-Hex:5; HexNAc:5; Neu5Ac:1; @sulfate:1; a,en-Hex:1} GAG-Linker

{Xyl:1; Hex:2; a-Hex:6; HexNAc:6; Neu5Ac:1; @sulfate:1; a,en-Hex:1} GAG-Linker

{Xyl:1; Hex:2; a-Hex:7; HexNAc:7; Neu5Ac:1; @sulfate:1; a,en-Hex:1} GAG-Linker

{Xyl:1; Hex:2; a-Hex:8; HexNAc:8; Neu5Ac:1; @sulfate:1; a,en-Hex:1} GAG-Linker

{Xyl:1; Hex:2; a-Hex:2; HexNAc:2; Neu5Gc:1; @sulfate:1; a,en-Hex:1} GAG-Linker

{Xyl:1; Hex:2; a-Hex:3; HexNAc:3; Neu5Gc:1; @sulfate:1; a,en-Hex:1} GAG-Linker

{Xyl:1; Hex:2; a-Hex:4; HexNAc:4; Neu5Gc:1; @sulfate:1; a,en-Hex:1} GAG-Linker

{Xyl:1; Hex:2; a-Hex:5; HexNAc:5; Neu5Gc:1; @sulfate:1; a,en-Hex:1} GAG-Linker

{Xyl:1; Hex:2; a-Hex:6; HexNAc:6; Neu5Gc:1; @sulfate:1; a,en-Hex:1} GAG-Linker

{Xyl:1; Hex:2; a-Hex:7; HexNAc:7; Neu5Gc:1; @sulfate:1; a,en-Hex:1} GAG-Linker

{Xyl:1; Hex:2; a-Hex:8; HexNAc:8; Neu5Gc:1; @sulfate:1; a,en-Hex:1} GAG-Linker

{XylP:1; Hex:2; a-Hex:2; HexNAc:2; Neu5Ac:1; @sulfate:1; a,en-Hex:1} GAG-Linker

{XylP:1; Hex:2; a-Hex:3; HexNAc:3; Neu5Ac:1; @sulfate:1; a,en-Hex:1} GAG-Linker

{XylP:1; Hex:2; a-Hex:4; HexNAc:4; Neu5Ac:1; @sulfate:1; a,en-Hex:1} GAG-Linker

{XylP:1; Hex:2; a-Hex:5; HexNAc:5; Neu5Ac:1; @sulfate:1; a,en-Hex:1} GAG-Linker

{XylP:1; Hex:2; a-Hex:6; HexNAc:6; Neu5Ac:1; @sulfate:1; a,en-Hex:1} GAG-Linker

{XylP:1; Hex:2; a-Hex:7; HexNAc:7; Neu5Ac:1; @sulfate:1; a,en-Hex:1} GAG-Linker

{XylP:1; Hex:2; a-Hex:8; HexNAc:8; Neu5Ac:1; @sulfate:1; a,en-Hex:1} GAG-Linker

{XylP:1; Hex:2; a-Hex:2; HexNAc:2; Neu5Gc:1; @sulfate:1; a,en-Hex:1} GAG-Linker

{XylP:1; Hex:2; a-Hex:3; HexNAc:3; Neu5Gc:1; @sulfate:1; a,en-Hex:1} GAG-Linker

{XylP:1; Hex:2; a-Hex:4; HexNAc:4; Neu5Gc:1; @sulfate:1; a,en-Hex:1} GAG-Linker

{XylP:1; Hex:2; a-Hex:5; HexNAc:5; Neu5Gc:1; @sulfate:1; a,en-Hex:1} GAG-Linker

{XylP:1; Hex:2; a-Hex:6; HexNAc:6; Neu5Gc:1; @sulfate:1; a,en-Hex:1} GAG-Linker

{XylP:1; Hex:2; a-Hex:7; HexNAc:7; Neu5Gc:1; @sulfate:1; a,en-Hex:1} GAG-Linker

{XylP:1; Hex:2; a-Hex:8; HexNAc:8; Neu5Gc:1; @sulfate:1; a,en-Hex:1} GAG-Linker

{Xyl:1; Hex:2; a-Hex:2; HexNAc:2; @sulfate:2; a,en-Hex:1} GAG-Linker

{Xyl:1; Hex:2; a-Hex:3; HexNAc:3; @sulfate:2; a,en-Hex:1} GAG-Linker

{Xyl:1; Hex:2; a-Hex:4; HexNAc:4; @sulfate:2; a,en-Hex:1} GAG-Linker

{Xyl:1; Hex:2; a-Hex:5; HexNAc:5; @sulfate:2; a,en-Hex:1} GAG-Linker

{Xyl:1; Hex:2; a-Hex:6; HexNAc:6; @sulfate:2; a,en-Hex:1} GAG-Linker

{Xyl:1; Hex:2; a-Hex:7; HexNAc:7; @sulfate:2; a,en-Hex:1} GAG-Linker

{Xyl:1; Hex:2; a-Hex:8; HexNAc:8; @sulfate:2; a,en-Hex:1} GAG-Linker

{XylP:1; Hex:2; a-Hex:2; HexNAc:2; @sulfate:2; a,en-Hex:1} GAG-Linker

{XylP:1; Hex:2; a-Hex:3; HexNAc:3; @sulfate:2; a,en-Hex:1} GAG-Linker

{XylP:1; Hex:2; a-Hex:4; HexNAc:4; @sulfate:2; a,en-Hex:1} GAG-Linker

{XylP:1; Hex:2; a-Hex:5; HexNAc:5; @sulfate:2; a,en-Hex:1} GAG-Linker

{XylP:1; Hex:2; a-Hex:6; HexNAc:6; @sulfate:2; a,en-Hex:1} GAG-Linker

{XylP:1; Hex:2; a-Hex:7; HexNAc:7; @sulfate:2; a,en-Hex:1} GAG-Linker

{XylP:1; Hex:2; a-Hex:8; HexNAc:8; @sulfate:2; a,en-Hex:1} GAG-Linker

{Xyl:1; Hex:2; a-Hex:2; HexNAc:2; Neu5Ac:1; @sulfate:2; a,en-Hex:1} GAG-Linker

{Xyl:1; Hex:2; a-Hex:3; HexNAc:3; Neu5Ac:1; @sulfate:2; a,en-Hex:1} GAG-Linker

{Xyl:1; Hex:2; a-Hex:4; HexNAc:4; Neu5Ac:1; @sulfate:2; a,en-Hex:1} GAG-Linker

{Xyl:1; Hex:2; a-Hex:5; HexNAc:5; Neu5Ac:1; @sulfate:2; a,en-Hex:1} GAG-Linker

{Xyl:1; Hex:2; a-Hex:6; HexNAc:6; Neu5Ac:1; @sulfate:2; a,en-Hex:1} GAG-Linker

{Xyl:1; Hex:2; a-Hex:7; HexNAc:7; Neu5Ac:1; @sulfate:2; a,en-Hex:1} GAG-Linker

{Xyl:1; Hex:2; a-Hex:8; HexNAc:8; Neu5Ac:1; @sulfate:2; a,en-Hex:1} GAG-Linker

{Xyl:1; Hex:2; a-Hex:2; HexNAc:2; Neu5Gc:1; @sulfate:2; a,en-Hex:1} GAG-Linker

{Xyl:1; Hex:2; a-Hex:3; HexNAc:3; Neu5Gc:1; @sulfate:2; a,en-Hex:1} GAG-Linker

{Xyl:1; Hex:2; a-Hex:4; HexNAc:4; Neu5Gc:1; @sulfate:2; a,en-Hex:1} GAG-Linker

{Xyl:1; Hex:2; a-Hex:5; HexNAc:5; Neu5Gc:1; @sulfate:2; a,en-Hex:1} GAG-Linker

{Xyl:1; Hex:2; a-Hex:6; HexNAc:6; Neu5Gc:1; @sulfate:2; a,en-Hex:1} GAG-Linker

{Xyl:1; Hex:2; a-Hex:7; HexNAc:7; Neu5Gc:1; @sulfate:2; a,en-Hex:1} GAG-Linker

{Xyl:1; Hex:2; a-Hex:8; HexNAc:8; Neu5Gc:1; @sulfate:2; a,en-Hex:1} GAG-Linker

{XylP:1; Hex:2; a-Hex:2; HexNAc:2; Neu5Ac:1; @sulfate:2; a,en-Hex:1} GAG-Linker

{XylP:1; Hex:2; a-Hex:3; HexNAc:3; Neu5Ac:1; @sulfate:2; a,en-Hex:1} GAG-Linker

{XylP:1; Hex:2; a-Hex:4; HexNAc:4; Neu5Ac:1; @sulfate:2; a,en-Hex:1} GAG-Linker

{XylP:1; Hex:2; a-Hex:5; HexNAc:5; Neu5Ac:1; @sulfate:2; a,en-Hex:1} GAG-Linker

{XylP:1; Hex:2; a-Hex:6; HexNAc:6; Neu5Ac:1; @sulfate:2; a,en-Hex:1} GAG-Linker

{XylP:1; Hex:2; a-Hex:7; HexNAc:7; Neu5Ac:1; @sulfate:2; a,en-Hex:1} GAG-Linker

{XylP:1; Hex:2; a-Hex:8; HexNAc:8; Neu5Ac:1; @sulfate:2; a,en-Hex:1} GAG-Linker

{XylP:1; Hex:2; a-Hex:2; HexNAc:2; Neu5Gc:1; @sulfate:2; a,en-Hex:1} GAG-Linker

{XylP:1; Hex:2; a-Hex:3; HexNAc:3; Neu5Gc:1; @sulfate:2; a,en-Hex:1} GAG-Linker

{XylP:1; Hex:2; a-Hex:4; HexNAc:4; Neu5Gc:1; @sulfate:2; a,en-Hex:1} GAG-Linker

{XylP:1; Hex:2; a-Hex:5; HexNAc:5; Neu5Gc:1; @sulfate:2; a,en-Hex:1} GAG-Linker

{XylP:1; Hex:2; a-Hex:6; HexNAc:6; Neu5Gc:1; @sulfate:2; a,en-Hex:1} GAG-Linker

{XylP:1; Hex:2; a-Hex:7; HexNAc:7; Neu5Gc:1; @sulfate:2; a,en-Hex:1} GAG-Linker

{XylP:1; Hex:2; a-Hex:8; HexNAc:8; Neu5Gc:1; @sulfate:2; a,en-Hex:1} GAG-Linker

{Xyl:1; Hex:2; a-Hex:2; HexNAc:2; @sulfate:3; a,en-Hex:1} GAG-Linker

{Xyl:1; Hex:2; a-Hex:3; HexNAc:3; @sulfate:3; a,en-Hex:1} GAG-Linker

{Xyl:1; Hex:2; a-Hex:4; HexNAc:4; @sulfate:3; a,en-Hex:1} GAG-Linker

{Xyl:1; Hex:2; a-Hex:5; HexNAc:5; @sulfate:3; a,en-Hex:1} GAG-Linker

{Xyl:1; Hex:2; a-Hex:6; HexNAc:6; @sulfate:3; a,en-Hex:1} GAG-Linker

{Xyl:1; Hex:2; a-Hex:7; HexNAc:7; @sulfate:3; a,en-Hex:1} GAG-Linker

{Xyl:1; Hex:2; a-Hex:8; HexNAc:8; @sulfate:3; a,en-Hex:1} GAG-Linker

{XylP:1; Hex:2; a-Hex:2; HexNAc:2; @sulfate:3; a,en-Hex:1} GAG-Linker

{XylP:1; Hex:2; a-Hex:3; HexNAc:3; @sulfate:3; a,en-Hex:1} GAG-Linker

{XylP:1; Hex:2; a-Hex:4; HexNAc:4; @sulfate:3; a,en-Hex:1} GAG-Linker

{XylP:1; Hex:2; a-Hex:5; HexNAc:5; @sulfate:3; a,en-Hex:1} GAG-Linker

{XylP:1; Hex:2; a-Hex:6; HexNAc:6; @sulfate:3; a,en-Hex:1} GAG-Linker

{XylP:1; Hex:2; a-Hex:7; HexNAc:7; @sulfate:3; a,en-Hex:1} GAG-Linker

{XylP:1; Hex:2; a-Hex:8; HexNAc:8; @sulfate:3; a,en-Hex:1} GAG-Linker

{Xyl:1; Hex:2; a-Hex:2; HexNAc:2; Neu5Ac:1; @sulfate:3; a,en-Hex:1} GAG-Linker

{Xyl:1; Hex:2; a-Hex:3; HexNAc:3; Neu5Ac:1; @sulfate:3; a,en-Hex:1} GAG-Linker

{Xyl:1; Hex:2; a-Hex:4; HexNAc:4; Neu5Ac:1; @sulfate:3; a,en-Hex:1} GAG-Linker

{Xyl:1; Hex:2; a-Hex:5; HexNAc:5; Neu5Ac:1; @sulfate:3; a,en-Hex:1} GAG-Linker

{Xyl:1; Hex:2; a-Hex:6; HexNAc:6; Neu5Ac:1; @sulfate:3; a,en-Hex:1} GAG-Linker

{Xyl:1; Hex:2; a-Hex:7; HexNAc:7; Neu5Ac:1; @sulfate:3; a,en-Hex:1} GAG-Linker

{Xyl:1; Hex:2; a-Hex:8; HexNAc:8; Neu5Ac:1; @sulfate:3; a,en-Hex:1} GAG-Linker

{Xyl:1; Hex:2; a-Hex:2; HexNAc:2; Neu5Gc:1; @sulfate:3; a,en-Hex:1} GAG-Linker

{Xyl:1; Hex:2; a-Hex:3; HexNAc:3; Neu5Gc:1; @sulfate:3; a,en-Hex:1} GAG-Linker

{Xyl:1; Hex:2; a-Hex:4; HexNAc:4; Neu5Gc:1; @sulfate:3; a,en-Hex:1} GAG-Linker

{Xyl:1; Hex:2; a-Hex:5; HexNAc:5; Neu5Gc:1; @sulfate:3; a,en-Hex:1} GAG-Linker

{Xyl:1; Hex:2; a-Hex:6; HexNAc:6; Neu5Gc:1; @sulfate:3; a,en-Hex:1} GAG-Linker

{Xyl:1; Hex:2; a-Hex:7; HexNAc:7; Neu5Gc:1; @sulfate:3; a,en-Hex:1} GAG-Linker

{Xyl:1; Hex:2; a-Hex:8; HexNAc:8; Neu5Gc:1; @sulfate:3; a,en-Hex:1} GAG-Linker

{XylP:1; Hex:2; a-Hex:2; HexNAc:2; Neu5Ac:1; @sulfate:3; a,en-Hex:1} GAG-Linker

{XylP:1; Hex:2; a-Hex:3; HexNAc:3; Neu5Ac:1; @sulfate:3; a,en-Hex:1} GAG-Linker

{XylP:1; Hex:2; a-Hex:4; HexNAc:4; Neu5Ac:1; @sulfate:3; a,en-Hex:1} GAG-Linker

{XylP:1; Hex:2; a-Hex:5; HexNAc:5; Neu5Ac:1; @sulfate:3; a,en-Hex:1} GAG-Linker

{XylP:1; Hex:2; a-Hex:6; HexNAc:6; Neu5Ac:1; @sulfate:3; a,en-Hex:1} GAG-Linker

{XylP:1; Hex:2; a-Hex:7; HexNAc:7; Neu5Ac:1; @sulfate:3; a,en-Hex:1} GAG-Linker

{XylP:1; Hex:2; a-Hex:8; HexNAc:8; Neu5Ac:1; @sulfate:3; a,en-Hex:1} GAG-Linker

{XylP:1; Hex:2; a-Hex:2; HexNAc:2; Neu5Gc:1; @sulfate:3; a,en-Hex:1} GAG-Linker

{XylP:1; Hex:2; a-Hex:3; HexNAc:3; Neu5Gc:1; @sulfate:3; a,en-Hex:1} GAG-Linker

{XylP:1; Hex:2; a-Hex:4; HexNAc:4; Neu5Gc:1; @sulfate:3; a,en-Hex:1} GAG-Linker

{XylP:1; Hex:2; a-Hex:5; HexNAc:5; Neu5Gc:1; @sulfate:3; a,en-Hex:1} GAG-Linker

{XylP:1; Hex:2; a-Hex:6; HexNAc:6; Neu5Gc:1; @sulfate:3; a,en-Hex:1} GAG-Linker

{XylP:1; Hex:2; a-Hex:7; HexNAc:7; Neu5Gc:1; @sulfate:3; a,en-Hex:1} GAG-Linker

{XylP:1; Hex:2; a-Hex:8; HexNAc:8; Neu5Gc:1; @sulfate:3; a,en-Hex:1} GAG-Linker

{Xyl:1; Hex:2; a-Hex:2; HexNAc:2; @sulfate:4; a,en-Hex:1} GAG-Linker

{Xyl:1; Hex:2; a-Hex:3; HexNAc:3; @sulfate:4; a,en-Hex:1} GAG-Linker

{Xyl:1; Hex:2; a-Hex:4; HexNAc:4; @sulfate:4; a,en-Hex:1} GAG-Linker

{Xyl:1; Hex:2; a-Hex:5; HexNAc:5; @sulfate:4; a,en-Hex:1} GAG-Linker

{Xyl:1; Hex:2; a-Hex:6; HexNAc:6; @sulfate:4; a,en-Hex:1} GAG-Linker

{Xyl:1; Hex:2; a-Hex:7; HexNAc:7; @sulfate:4; a,en-Hex:1} GAG-Linker

{Xyl:1; Hex:2; a-Hex:8; HexNAc:8; @sulfate:4; a,en-Hex:1} GAG-Linker

{XylP:1; Hex:2; a-Hex:2; HexNAc:2; @sulfate:4; a,en-Hex:1} GAG-Linker

{XylP:1; Hex:2; a-Hex:3; HexNAc:3; @sulfate:4; a,en-Hex:1} GAG-Linker

{XylP:1; Hex:2; a-Hex:4; HexNAc:4; @sulfate:4; a,en-Hex:1} GAG-Linker

{XylP:1; Hex:2; a-Hex:5; HexNAc:5; @sulfate:4; a,en-Hex:1} GAG-Linker

{XylP:1; Hex:2; a-Hex:6; HexNAc:6; @sulfate:4; a,en-Hex:1} GAG-Linker

{XylP:1; Hex:2; a-Hex:7; HexNAc:7; @sulfate:4; a,en-Hex:1} GAG-Linker

{XylP:1; Hex:2; a-Hex:8; HexNAc:8; @sulfate:4; a,en-Hex:1} GAG-Linker

{Xyl:1; Hex:2; a-Hex:2; HexNAc:2; Neu5Ac:1; @sulfate:4; a,en-Hex:1} GAG-Linker

{Xyl:1; Hex:2; a-Hex:3; HexNAc:3; Neu5Ac:1; @sulfate:4; a,en-Hex:1} GAG-Linker

{Xyl:1; Hex:2; a-Hex:4; HexNAc:4; Neu5Ac:1; @sulfate:4; a,en-Hex:1} GAG-Linker

{Xyl:1; Hex:2; a-Hex:5; HexNAc:5; Neu5Ac:1; @sulfate:4; a,en-Hex:1} GAG-Linker

{Xyl:1; Hex:2; a-Hex:6; HexNAc:6; Neu5Ac:1; @sulfate:4; a,en-Hex:1} GAG-Linker

{Xyl:1; Hex:2; a-Hex:7; HexNAc:7; Neu5Ac:1; @sulfate:4; a,en-Hex:1} GAG-Linker

{Xyl:1; Hex:2; a-Hex:8; HexNAc:8; Neu5Ac:1; @sulfate:4; a,en-Hex:1} GAG-Linker

{Xyl:1; Hex:2; a-Hex:2; HexNAc:2; Neu5Gc:1; @sulfate:4; a,en-Hex:1} GAG-Linker

{Xyl:1; Hex:2; a-Hex:3; HexNAc:3; Neu5Gc:1; @sulfate:4; a,en-Hex:1} GAG-Linker

{Xyl:1; Hex:2; a-Hex:4; HexNAc:4; Neu5Gc:1; @sulfate:4; a,en-Hex:1} GAG-Linker

{Xyl:1; Hex:2; a-Hex:5; HexNAc:5; Neu5Gc:1; @sulfate:4; a,en-Hex:1} GAG-Linker

{Xyl:1; Hex:2; a-Hex:6; HexNAc:6; Neu5Gc:1; @sulfate:4; a,en-Hex:1} GAG-Linker

{Xyl:1; Hex:2; a-Hex:7; HexNAc:7; Neu5Gc:1; @sulfate:4; a,en-Hex:1} GAG-Linker

{Xyl:1; Hex:2; a-Hex:8; HexNAc:8; Neu5Gc:1; @sulfate:4; a,en-Hex:1} GAG-Linker

{XylP:1; Hex:2; a-Hex:2; HexNAc:2; Neu5Ac:1; @sulfate:4; a,en-Hex:1} GAG-Linker

{XylP:1; Hex:2; a-Hex:3; HexNAc:3; Neu5Ac:1; @sulfate:4; a,en-Hex:1} GAG-Linker

{XylP:1; Hex:2; a-Hex:4; HexNAc:4; Neu5Ac:1; @sulfate:4; a,en-Hex:1} GAG-Linker

{XylP:1; Hex:2; a-Hex:5; HexNAc:5; Neu5Ac:1; @sulfate:4; a,en-Hex:1} GAG-Linker

{XylP:1; Hex:2; a-Hex:6; HexNAc:6; Neu5Ac:1; @sulfate:4; a,en-Hex:1} GAG-Linker

{XylP:1; Hex:2; a-Hex:7; HexNAc:7; Neu5Ac:1; @sulfate:4; a,en-Hex:1} GAG-Linker

{XylP:1; Hx:2; a-Hex:8; HexNAc:8; Neu5Ac:1; @sulfate:4; a,en-Hex:1} GAG-Linker

{XylP:1; Hex:2; a-Hex:2; HexNAc:2; Neu5Gc:1; @sulfate:4; a,en-Hex:1} GAG-Linker

{XylP:1; Hex:2; a-Hex:3; HexNAc:3; Neu5Gc:1; @sulfate:4; a,en-Hex:1} GAG-Linker

{XylP:1; Hex:2; a-Hex:4; HexNAc:4; Neu5Gc:1; @sulfate:4; a,en-Hex:1} GAG-Linker

{XylP:1; Hex:2; a-Hex:5; HexNAc:5; Neu5Gc:1; @sulfate:4; a,en-Hex:1} GAG-Linker

{XylP:1; Hex:2; a-Hex:6; HexNAc:6; Neu5Gc:1; @sulfate:4; a,en-Hex:1} GAG-Linker

{XylP:1; Hex:2; a-Hex:7; HexNAc:7; Neu5Gc:1; @sulfate:4; a,en-Hex:1} GAG-Linker

{XylP:1; Hex:2; a-Hex:8; HexNAc:8; Neu5Gc:1; @sulfate:4; a,en-Hex:1} GAG-Linker

{Fuc:1; Hex:3; HexNAc:3; Neu5Ac:1} O-Glycan

{Hex:1; HexNAc:1; Neu5Ac:2} O-Glycan

{Fuc:1; Hex:2; HexNAc:3; Neu5Ac:1} O-Glycan

{Fuc:2; Hex:2; HexNAc:2; Neu5Ac:1} O-Glycan

{Hex:2; HexNAc:2} O-Glycan

{Fuc:1; Hex:1; HexNAc:2} O-Glycan

{Fuc:1; Hex:2; HexNAc:3} O-Glycan

{Hex:1; HexNAc:1; Neu5Ac:1} O-Glycan

{Fuc:1; Hex:2; HexNAc:2} O-Glycan

{Fuc:1; Hex:3; HexNAc:3} O-Glycan

{Hex:3; HexNAc:3} O-Glycan

{Fuc:1; Hex:4; HexNAc:4; Neu5Ac:1} O-Glycan

{Hex:4; HexNAc:4} O-Glycan

{Fuc:1; Hex:4; HexNAc:4} O-Glycan

{Fuc:1; Hex:1; HexNAc:2; Neu5Ac:2} O-Glycan

{Fuc:1; Hex:1; HexNAc:3; Neu5Ac:1} O-Glycan

{HexNAc:1} O-Glycan

{Hex:3; HexNAc:5} O-Glycan

{Fuc:1; Hex:3; HexNAc:4} O-Glycan

{Fuc:1; Hex:4; HexNAc:4; Neu5Ac:2} O-Glycan

{Hex:3; HexNAc:7; Neu5Ac:1} O-Glycan

{Hex:4; HexNAc:7} O-Glycan

{Hex:3; HexNAc:8} O-Glycan

{HexNAc:2; Neu5Ac:1} O-Glycan

{Fuc:2; Hex:2; HexNAc:3; Neu5Ac:1} O-Glycan

{Fuc:1; Hex:2; HexNAc:2; Neu5Ac:2} O-Glycan

{Fuc:1; Hex:4; HexNAc:5; Neu5Ac:1} O-Glycan

{Hex:2; HexNAc:6} O-Glycan

{Fuc:1; Hex:3; HexNAc:7} O-Glycan
